# L-arginine-loaded microneedle patch enhances diabetic wound healing by regulating macrophage polarisation and mitochondrial homeostasis

**DOI:** 10.1093/rb/rbaf092

**Published:** 2025-09-01

**Authors:** Hong Wang, Shun Yao, Qingyun Mo, Mingyue Chen, Danfeng He, Lingfeng Yan, Chang Wang, Tao Zou, Gaoxing Luo, Jun Deng

**Affiliations:** Institute of Burn Research, State Key Laboratory of Trauma and Chemical Poisoning, The First Affiliated Hospital of Army Medical University (The Third Military Medical University), Chongqing 400038, China; Institute of Burn Research, State Key Laboratory of Trauma and Chemical Poisoning, The First Affiliated Hospital of Army Medical University (The Third Military Medical University), Chongqing 400038, China; Institute of Burn Research, State Key Laboratory of Trauma and Chemical Poisoning, The First Affiliated Hospital of Army Medical University (The Third Military Medical University), Chongqing 400038, China; School of Medicine, Southeast University, Nanjing, 210009, China; College of Bioengineering, Chongqing University, Chongqing, 400044, China; Institute of Burn Research, State Key Laboratory of Trauma and Chemical Poisoning, The First Affiliated Hospital of Army Medical University (The Third Military Medical University), Chongqing 400038, China; Institute of Burn Research, State Key Laboratory of Trauma and Chemical Poisoning, The First Affiliated Hospital of Army Medical University (The Third Military Medical University), Chongqing 400038, China; Institute of Burn Research, State Key Laboratory of Trauma and Chemical Poisoning, The First Affiliated Hospital of Army Medical University (The Third Military Medical University), Chongqing 400038, China; Institute of Burn Research, State Key Laboratory of Trauma and Chemical Poisoning, The First Affiliated Hospital of Army Medical University (The Third Military Medical University), Chongqing 400038, China; Institute of Burn Research, State Key Laboratory of Trauma and Chemical Poisoning, The First Affiliated Hospital of Army Medical University (The Third Military Medical University), Chongqing 400038, China; Institute of Burn Research, State Key Laboratory of Trauma and Chemical Poisoning, The First Affiliated Hospital of Army Medical University (The Third Military Medical University), Chongqing 400038, China

**Keywords:** microneedle, L-arginine, oxidative stress, mitochondrial homeostasis, macrophage polarization

## Abstract

Excessive oxidative stress and dysregulated macrophage polarization—characterized by M1/M2 imbalance—drive chronic, persistent inflammation and represent key pathological mechanisms underlying impaired tissue repair in diabetic wounds; however, therapeutic strategies targeting both these processes remain limited. L-arginine (L-Arg) shows therapeutic potential through its antioxidant properties and ability to promote M1 macrophage polarization. Nevertheless, the mechanisms by which L-Arg regulates mitochondrial homeostasis to exert antioxidant effects remain unclear. Moreover, its clinical translation is hindered by poor retention, inadequate tissue penetration and damage induced by hypertonicity, thereby necessitating the development of innovative delivery systems. To address these limitations, we developed an L-Arg-loaded microneedle (L-Arg-MN) patch for controlled delivery. Our findings demonstrate that L-Arg alleviated hydrogen peroxide (H_2_O_2_)-induced cellular damage through activation of the Kelch-like ECH-associated protein 1 (KEAP1)–nuclear factor erythroid 2-related factor 2 (Nrf2)–heme oxygenase-1 (HO-1) pathway, boosting antioxidant enzyme (superoxide dismutase (SOD), catalase (CAT) and glutathione peroxidase (GSH-Px)) and lowering malondialdehyde (MDA) levels. Mechanistically, L-Arg maintained mitochondrial homeostasis by upregulating peroxiredoxin 1 (PRDX1) expression, restoring mitochondrial membrane potential and enhancing adenosine triphosphate production. Furthermore, L-Arg suppressed M1 macrophage polarization and promoted M2 polarization through PRDX1-mediated mitochondrial metabolic pathways. In models of diabetic wounds, the L-Arg-MN patch markedly enhanced the wound healing process, accelerated wound closure, reduced concentration of reactive oxygen species (ROS), enhanced granulation tissue, collagen formation and increased M2 macrophage infiltration. This study elucidates how L-Arg reduces oxidative stress and enhances M2 macrophage polarization by regulating mitochondrial metabolism through the PRDX1 pathway. By integrating the metabolic and immunomodulatory properties of L-Arg with advanced drug delivery technology, the L-Arg-MN patch presents an innovative and efficient approach to treating diabetic wounds.

## Graphical abstract

**Figure:**
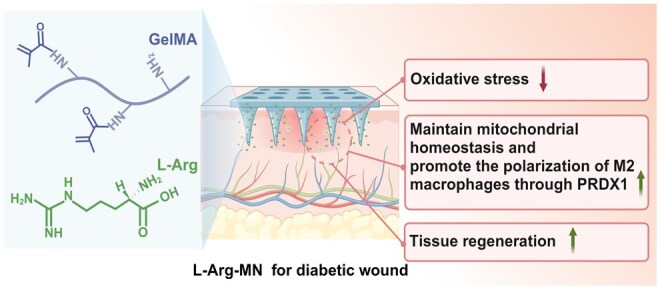

## Introduction

Diabetic foot ulcers (DFUs) are one of the most grave complications associated with diabetes, characterized by high rates of intractability, disability, mortality and recurrence [1, 2]. The poor healing capacity of DFUs is mainly due to chronic inflammation resulting from an imbalance in macrophage polarization (difficulty in transforming from the pro-inflammatory M1 to the anti-inflammatory M2 phenotype) and elevated oxidative stress [3]. Recent studies highlight the key influence of mitochondrial metabolism in driving macrophage functional changes [3, 4]. Notably, the overproduction of reactive oxygen species (ROS) leads to mitochondrial dysfunction, establishing a harmful cycle that perpetuates inflammation and oxidative stress, further hindering wound healing [5]. Conventional monotherapies that target either isolated inflammatory or oxidative pathways have shown limited efficacy, underscoring the need for dual-pathway strategies that simultaneously address macrophage polarization imbalance and oxidative damage to disrupt this cycle.

Recent studies have emphasized the critical role of amino acid metabolism [6, 7], specifically L-arginine (L-Arg), in maintaining a delicate balance between oxidative homeostasis and the resolution of inflammation through its multifaceted biological actions [8]. L-Arg is recognized as a central regulator bridging metabolic and immune reprogramming [9]. Previous research has shown that the AKT-Nrf2 and Nrf2-HO-1 signaling pathways are crucial for mitigating oxidative stress [10, 11]. These pathways enhance adenosine triphosphate (ATP) synthesis and reduce nitro-oxidative damage, thus preserving mitochondrial integrity [12–14]. Additionally, PRDX1 has been found to regulate macrophage polarization by maintaining mitochondrial homeostasis; its knockout results in increased oxidative damage and altered energy metabolism in macrophages [15, 16]. Interestingly, nanoparticles modified with L-Arg can specifically target M1 macrophages exhibiting upregulated expression of the cationic amino acid transporter protein 2, enabling precise delivery to inflammatory sites [17]. This underscores the critical role and therapeutic promise of L-Arg in macrophage polarization and targeted drug delivery. However, it remains unclear whether L-Arg can modulate mitochondrial homeostasis through PRDX1. Moreover, conventional therapeutic approaches, such as the delivery of antioxidants or cytokines through nanocarriers [18, 19], have been explored to regulate macrophage polarization, but they often yield only short-term effects and fail to address mitochondrial dysfunction. Similarly, conventional drug delivery strategies, such as subcutaneous injections and hydrogel dressings, may be hampered by issues, such as rapid drug release, inadequate tissue penetration and inconsistent drug concentrations [20, 21]. Therefore, there is a critical need to develop innovative wound-healing biomaterials that can regulate macrophage mitochondrial homeostasis through amino acid metabolism while enabling sustained and controlled drug delivery to enhance tissue repair.

**Figure 1.**
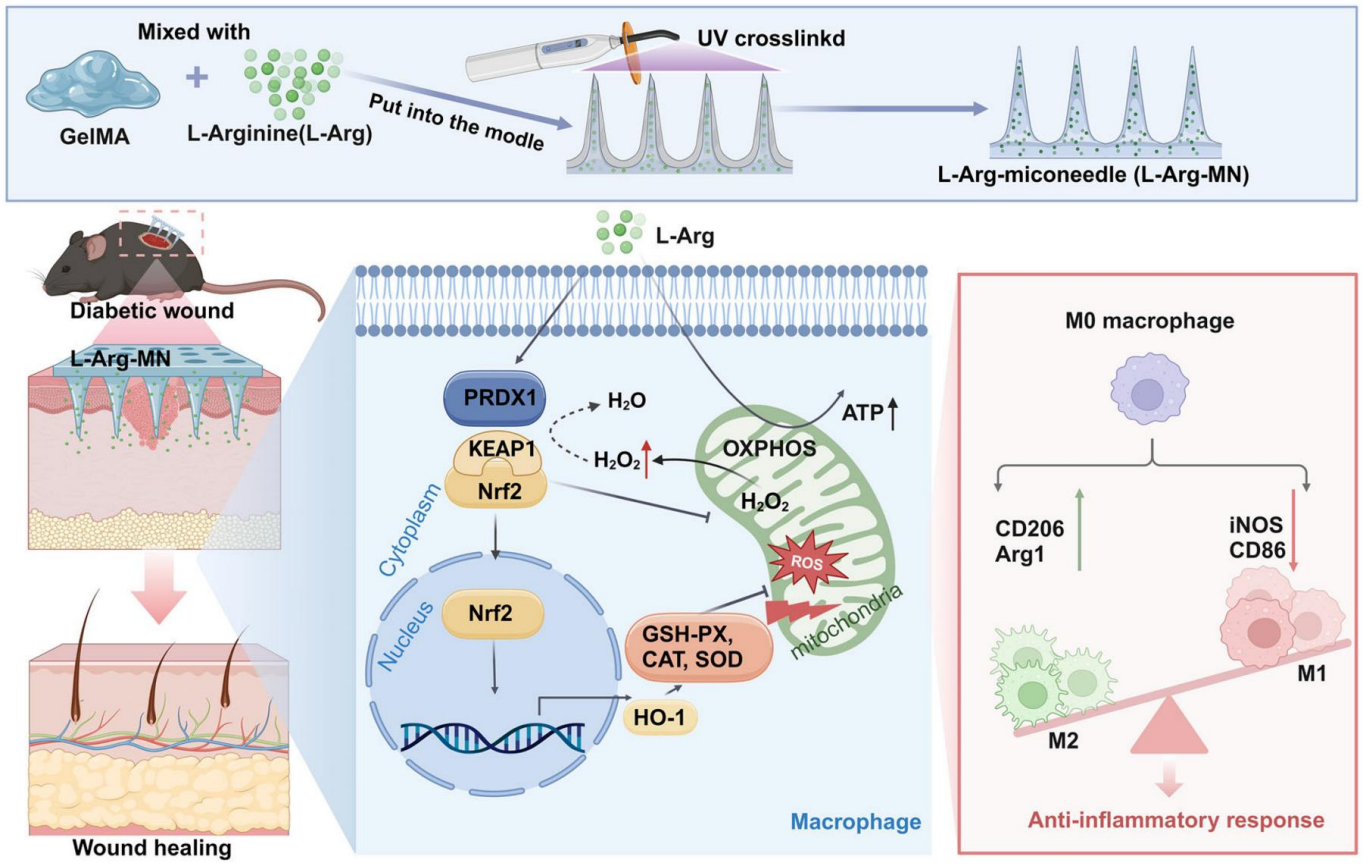
Schematic of how L-Arg-MN mitigates oxidative stress, regulates inflammation and accelerates wound healing through the KEAP1–Nrf2–HO-1/PRDX1 signaling pathway (schematic illustrations were created using BioRender: www.biorender.com).

To address these limitations, our study developed a soluble microneedle patch (L-Arg-MN) using FDA-approved Gelatin Methacrylate (GelMA), a biocompatible material known for its safety and versatility in biomedical applications [22]. L-Arg, the amino acid with the highest basicity, possesses highly reactive amino groups (–NH2) that readily form hydrogen bonds with the methacryloyl groups in GelMA [22]. This interaction substantially enhances the stability of L-Arg within the GelMA matrix and facilitates its controlled release, thereby enhancing diabetic wound repair, as illustrated in Figure 1. The MN array facilitates the precise delivery of L-Arg to deep wound tissues through its physical penetration capability, effectively preventing hyperosmotic damage. We began by investigating the effects of L-Arg on macrophage polarization and mitochondrial function to shed light on the underlying mechanisms and therapeutic benefits of L-Arg in diabetic wound care. Our results showed that L-Arg maintains mitochondrial homeostasis by restoring the mitochondrial membrane potential (MMP) through PRDX1. This action not only reshapes macrophage energy metabolism but also promotes M2 macrophage polarization. Upregulate antioxidant enzyme levels through activation of the Kelch-like ECH-associated protein 1 (KEAP1)–nuclear factor erythroid 2-related factor 2 (Nrf2)–heme oxygenase-1 (HO-1) pathway. L-Arg effectively disrupts the ROS-inflammation cycle, thus accelerating wound healing in diabetic models. This dual regulation of metabolism and phenotype enhances M2 macrophage polarization, further accelerating wound healing in diabetic models. The L-Arg-MN patch offers a sustained and targeted delivery system, addressing the limitations of poor retention, hyperosmotic damage and limited deep tissue penetration associated with conventional drug delivery methods. In summary, this research puts forward a new therapeutic strategy for treating chronic wounds and offers significant insights into macrophage polarization-based therapeutic interventions. Additionally, a comprehensive analysis of the mechanisms underlying L-Arg’s activity is crucial for identifying novel therapeutic targets in antioxidant and immune-modulating treatments.

## Materials and methods

The Ethics Committee of the Army Military Medical University approved this study (Approval No. AMUWEC20237071), and all experiments adhered to the university's guidelines. The experiments were conducted in accordance with the guidelines of the Army Military Medical University and the relevant national authorities for the management and use of experimental animals.

### Chemicals and materials

All high-purity synthetic reagents were commercially sourced, including GelMA (CureGelCo, Ltd), lithium phenyl-2,4,6-trimethylbenzoylphosphinate (LAP, MedChemExpress), L-Arg (C_6_H_14_N_4_O_2_, Macklin), 1-naphthol (C_10_H_8_O, Macklin), 2,3-butanedione (C_4_H_6_O_2_, Macklin), sodium hydroxide (NaOH, Macklin) and n-propanol (C_3_H_8_O, Macklin). Hydrogen peroxide (H_2_O_2_, 3%) solution was obtained from Aladdin Reagent (China). L-NMMA (Beyotime, China). The Fibre Tissue RNA Extraction Kit was sourced from Qiagen (Germany), and TRIzol RNA Extraction Reagent from CST (USA). Real-time fluorescent quantitative RT-PCR primers were designed and synthesized by Beijing BGI Genomics (China). The Degenerate Genomic DNA Reverse Transcription Kit and TB Green Quantitative PCR Enzyme were purchased from Takara (Japan). The aseptic transparent surgical application was supplied by 3M (USA), and the confocal dish was acquired from Bioshark (China).

### Instrumentation

This study utilized the LSM780 Laser Scanning Confocal Microscope (Zeiss, Germany), CFX Connect Real-Time Fluorescent Quantitative PCR Instrument (Bio-Rad, USA) and a Digital Slide Scanner (Ningbo JiangFeng Bioinformation Technology, China). The microstructure of the porous MN patch was examined by SEM using a Hitachi SU8010. A VarioskanTM LUX from Thermo Fisher was used to measure the absorbance value of the solution.

### Preparation of gelatine methacrylate-loaded microneedle and L-Arg-loaded microneedle

GelMA (1 g, 5 wt%) and LAP (0.1 g, 0.5 wt%) were mixed into 20 ml of deionized water. About 500 μl of this solution was transferred into a negative mold and then placed under vacuum for 30 min to ensure that the MN holes were filled. The mold was subsequently put in an oven at 37°C for 10 h. After that, the GelMA hydrogel was solidified by UV light (365 nm, 100 W) for 30 s. The MN array was then gently removed from the negative mold to create a GelMA-loaded MN (GelMA-MN). For L-Arg-loaded GelMA-MN, the same procedure was followed.

### Detection of L-Arg from gelatine methacrylate-loaded microneedle

A chromogenic reagent was prepared by dissolving 1-naphthol (4% w/v) and diacetyl (20% w/v) in n-propanol (final volume 100 mL). For the analysis, 3 mL of the L-Arg sample was successively mixed with: (1) 0.5 mL of 14% NaOH, (2) 1.5 mL of the chromogenic reagent and (3) 1 mL of n-propanol. After an incubation period of 10 min at 40°C, the absorbance was measured at a wavelength of 530 nm.

### Cell culture and treatment

RAW 264.7 cells and NIH-3T3 mouse embryonic fibroblasts from ATCC were cultured in DMEM (Hyclone) supplemented with 10% FBS, at 37°C in the presence of 5% CO_2_, with daily medium change and passaging at 80–90% confluence.

### Cell viability assay

RAW 264.7 cells were seeded in 96-well plates at 8 × 10^5^ cells/mL, with 100 μL added to each well. After an overnight incubation, fresh medium supplemented with H_2_O_2_ (0–1000 μM) and L-Arg (0–32 mM) was added for 24 h. To examine the effect of L-Arg on cell survival under oxidative stress, cells were pretreated with L-Arg for 24 h, then exposed to 700 μM H_2_O_2_ for 6 h. The cells were then washed three times with PBS, incubated in medium containing CCK8 for 1–2 h. Absorbance was measured at 450 nm


$$\mathrm{Cell}\;\mathrm{survival}\;\mathrm{rate}\;(\%)=[\frac{\mathrm{As}-\mathrm{Ab}}{\mathrm{Ac}-\mathrm{Ab}}]\times 100\%.$$

Here, As denotes the absorbance of the sample, including CCK-8 solution with cells and either L-Arg or H_2_O_2_. Ac is the control absorbance (CCK-8 with cells but no L-Arg or H_2_O_2_), and Ab is the blank absorbance (CCK-8 solution without cells, L-Arg or H_2_O_2_).

### Detection of intracellular ROS

ROS are key markers of cellular oxidative stress [23]. Intracellular ROS levels were assessed using a commercial kit (Beyotime, China). Cells exposed to 200 μM H_2_O_2_ and/or 5 mM L-Arg for 24 h were incubated with 10 μM DCFH-DA, at 37°C for 30 min in the dark, then analysed via confocal microscopy with ImageJ quantification.

### Antioxidant enzyme activity and cellular ATP quantification

Following cell treatment, the activities of SOD (#BC0175, Solaibao, Beijing), CAT (#A007-1-1, Nanjing) and GSH (#BC1175, Solaibao, Beijing) were assessed, alongside measurements of MDA (S0131S, Beyotime, China) content and cellular ATP levels (S0027, Beyotime, China). All operations should be carried out in accordance with the instructions.

### Cellular immunofluorescence staining

RAW 264.7 cells were seeded on slides and incubated overnight. Cells were treated with 200 μM H_2_O_2_ and/or 5 mM L-Arg for 24 h, then fixed with 4% paraformaldehyde for 15 min and permeabilized with Triton X-100 for 10 min. Following blocking with goat serum, cells underwent incubation with primary antibodies KEAP1 (#8047, CST), Nrf2 (#12721, CST), PRDX1 (66820-1, Proteintech), iNOS (ab178945, Abcam), CD206 (ab64693, Abcam), Ki67 (ab16667, Abcam) and CD31 (ab222783, Abcam) overnight at 4°C. After PBS rinsing, cells were incubated with secondary antibodies or rhodamine phalloidin for 1 h in the dark. Coverslips were mounted with DAPI anti-fade reagent and imaged by confocal microscopy. Image analysis was done with ImageJ.

### Western blot

Cells lysed in RIPA buffer with inhibitors (Beyotime) were kept on ice. Wound tissues homogenized with a 5-mm grinder (Abcam) were centrifuged for supernatant collection. Protein concentrations were determined via BCA kit (Beyotime). Equal proteins were separated by SDS-PAGE and transferred to 0.22-µm PVDF membranes (Millipore). Membranes were blocked with 5% skim milk and incubated with primary antibodies GAPDH (#5174, CST), Histone H3 (#4499, CST), KEAP1 (#8047, CST), Nrf2 (#12721, CST), HO-1 (#43966, CST), PRDX1 (66820-1, Proteintech), iNOS (ab178945, Abcam) and CD206 (ab64693, Abcam) overnight at 4 °C. After incubation with a horseradish peroxidase-conjugated secondary antibody (1:3000; Beyotime) for 1 h, the membranes were developed. Protein band intensities were analysed using ImageJ software.

### Transfection assay

When RAW 264.7 cells reached 80% confluence, they were transfected with 50 nM PRDX1-targeting siRNA (Table 1) or non-targeting control siRNA using jetPRIME reagent. PRDX1 knockdown efficiency was assessed at the protein level after 48 h.

**Table 1.** siRNA sequences for target genes

Genes	Sense (5′-3′)	Anti-sense (3′–5′)
si-PRDX1	CCAGAUGGUCAGULUUAAAGAUTT	AUCUUUAAACUGACCAUCUGGTT
siNC	UUCUUCGAACGUGUCACGUTT	ACGUGACACGULUCGGAGAATT

### JC-1 staining

JC-1 is a widely used reagent for assessing MMP [24]. MMP was assessed using JC-1 (Beyotime). Cells were stained with JC-1 counterstained for 20 min and Hoechst (1:1000) for 10 min. Images captured using a confocal microscope.

### Mitochondrial ROS measurement

Mitochondrial superoxide levels were measured using MitoSOX™ Red (S0061S, Beyotime), a fluorescent probe specific to mitochondrial ROS [25]. Cells were incubated with 5 µM MitoSOX™ Red (37°C, 15 min), stained with Hoechst (1:1000, 10 min), captured using a confocal microscope.

### Determination of apoptosis

Apoptosis in RAW 264.7 cells was assessed using the Annexin V-FITC Apoptosis Detection Kit (C1062M, Beyotime). Incubated in the dark at room temperature for 20 min, after staining with Hoechst (1:1000, 10 min) in the dark on ice, fluorescent images were captured. The percentage of apoptotic cells was measured using ImageJ software.

### Macrophage polarization in vitro

RAW 264.7 cells were divided into four groups: blank control, LPS (200 ng/ml; Sigma) [26], treated with L-Arg (0.5 mM) or LPS + L-Arg, and all groups were cultured in DMEM for 24 h. For qRT-PCR, total RNA was extracted from RAW 264.7 cells using TRIzol reagent (Invitrogen). cDNA was synthesized with the PrimeScript™ Reverse Transcription Reagent Kit (TaKaRa). qPCR was performed using the SYBR^®^ Premix Ex Taq™ II kit (Takara), with primers listed in Table 2.

**Table 2.** Primer sequences used for quantitative real-time polymerase chain reaction

Genes	Direction	Primer sequence (5′–3′)
CD86	Forward	GGCTTGGCAATCCTTATCT
	Reverse	ACTTGGCATTCACACTATCA
CD206	Forward	TGGAAGAAGAAGTAGCCTATC
	Reverse	TGGAGTAGTGGTTGGAGAA
iNOS	Forward	TACTGCTGGTGGTGACAA
	Reverse	CTGAAGGTGTGGTTGAGTT
Arg-1	Forward	AAGGTCTCTACATCACAGAAG
	Reverse	CGAAGCAAGCCAAGGTTA
GAPDH	Forward	CTTTGTCAAGCTCATTTCCTGG
	Reverse	TCTTGCTCAGTGTCCTTGC

### Live/dead cell staining

The cytotoxicity of the hydrogel MN was evaluated using a Calcein AM/Propidium Iodide assay (C1371S, Beyotime). GelMA-MN and L-Arg-MN were co-cultured with 3T3 cells for 24–72 h. Stained with 10 µL each of calcein AM and propidium iodide for 10 min. Images captured using a confocal microscope.

### Construction of a diabetic mouse model

Male C57BL/6 mice (8–10 weeks old, 18–20 g) from the Army Medical University Animal Centre were used to establish a diabetic model with streptozotocin (STZ) and a high-fat, high-sugar diet[27]. Mice received 50 mg/kg STZ daily for 5 days, were fasted before injection and maintained on a high-fat, high-sugar diet. Mice with tail blood glucose levels ≥16.7 mmol/L for two consecutive weeks were considered to have Type 2 diabetes.

For the diabetic chronic wound healing experiment, diabetic mice were divided into three groups: blank control, L-Arg treatment and L-Arg-MN treatment. After anesthesia, full-thickness wounds (6 mm) were created on the back, and silicone rings were placed around them. Wounds were treated with PBS, L-Arg or L-Arg-MN, then covered with 3M dressings. Mice were monitored daily, and wounds were observed and photographed on days 0, 3, 7, 10 and 14. Wound areas were measured using ImageJ software to calculate healing rates. Healing rates were calculated using the following formula:


$$\mathrm{Wound}\;\mathrm{closure}\;\mathrm{rate}\;(\%)=\frac{S0-St}{S0}\times 100\%,$$

where *S*0 denotes the original wound area and *St* denotes the wound area at a given time point.

### Histological analysis

Wound tissue samples were collected for histological analysis. For H&E staining, samples were fixed, dehydrated, embedded and sectioned. Sections were deparaffinized in xylene, rehydrated in graded ethanol, stained with hematoxylin and eosin, then dehydrated and cleared with xylene. For cryosectioning, tissues were snap-frozen, cryoprotected in sucrose and embedded in OCT compound. Sections were stained with hematoxylin and processed as described. Sections were scanned and analysed using K-viewer software.

### Tissue ROS staining

On days 5 and 7, wound tissues were collected, snap-frozen in liquid nitrogen, cryoprotected in sucrose and embedded in OCT compound. After warming, sections were incubated with 10 μM DHE (MCE, USA) to assess ROS levels [28]. Sections were then sealed with an Antifade Mounting Medium containing DAPI.

### Immunofluorescence on paraffin sections

Paraffin-embedded wound tissues (from day 7 or 10) were deparaffinized, rehydrated and underwent antigen retrieval. Sections were permeabilized and blocked, then incubated overnight with primary antibodies (CD206, iNOS, Ki67, CD31; Abcam), (PRDX1; Proteintech) and secondary antibody, sealed with an anti-fluorescence quencher containing DAPI.Images were captured using a confocal microscope.

Protein interaction analysis was conducted using STRING (http://www.string-db.org/), GeneCards and PathCards.

### Statistical analysis

Results are displayed as mean ± SD based on at least three independent experiments. Statistical analysis was performed with GraphPad Prism 9.0. Multiple group comparisons were analysed by one-way ANOVA followed by Tukey’s *post hoc* test. Differences between the two groups were evaluated using a two-tailed unpaired Student’s *t*-test.

## Results

### L-Arg mitigates H_2_O_2_-induced cellular damage by enhancing antioxidant enzyme activity through the activation of the KEAP1–Nrf2–HO-1 pathway

**Figure 2.**
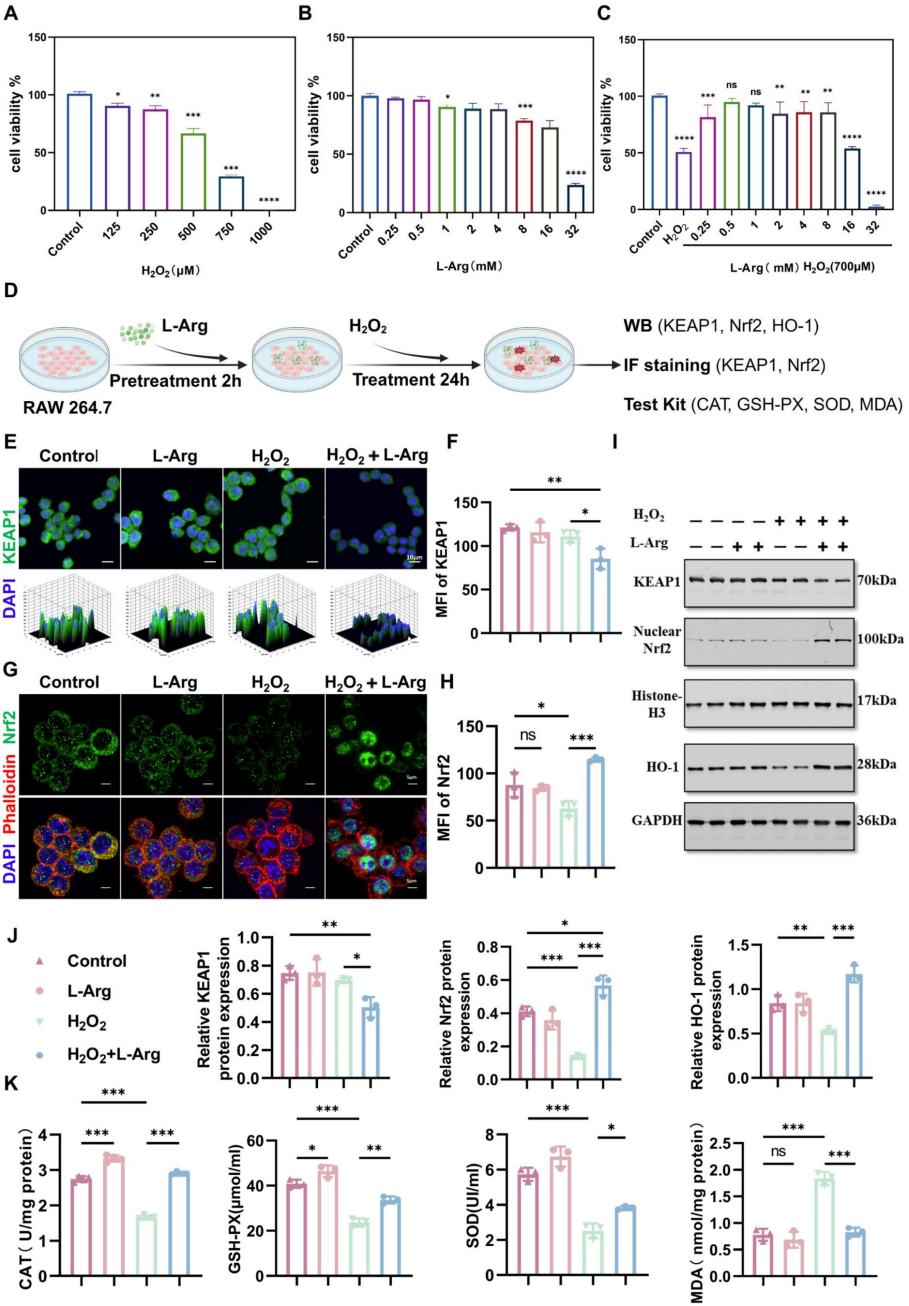
L-Arg attenuates H_2_O_2_-induced damage in RAW 264.7 macrophages by modulating the KEAP1–Nrf2–HO-1 pathway. (**A**) H_2_O_2_ dose–response (24 h); (**B**) L-Arg cytotoxicity (24 h); (**C**) L-Arg (0.25–32 mM, 2 h) pretreatment efficacy against 700 μM H_2_O_2_ (24 h); (**D**) Experimental schematic; (**E–H**) KEAP1/Nrf2 immunofluorescence with quantification; (**I, J**) Western blot of KEAP1, nuclear Nrf2 and HO-1; (**K**) Activities of antioxidant enzyme (CAT, GSH-Px, SOD) and MDA levels. Results are shown as mean ± SD from a minimum of three independent experiments. Statistical comparisons were performed using one-way ANOVA (*P < 0.05, **P < 0.01, ***P < 0.001; ns = not significant).

H_2_O_2_ induces cellular dysfunction and death by enhancing oxidative stress, impairing cellular components, activating apoptotic pathways and stimulating inflammatory responses [29]. Cellular viability directly reflects cell proliferation and survival. Co-culture with RAW 264.7 cells for 24 h showed that H_2_O_2_ induced cell death in a concentration-dependent manner, with concentrations ≥125 μM significantly reducing cell viability (Figure 2A). Cells treated with L-Arg for 24 h exhibited significantly reduced viability at 16 and 32 mM compared to the controls (Figure 2B). Pretreatment with L-Arg (0.25–32 mM) for 2 h, followed by 700 μM H_2_O_2_ exposure for 24 h, significantly mitigated H_2_O_2_-induced viability loss (Figure 2C). These results imply that L-Arg’s protective effect may be attributed to its antioxidant properties, possibly by activating the KEAP1–Nrf2–HO-1 pathway. Under typical physiological conditions, Nrf2 is situated in the cytoplasm, functioning as a component of the Nrf2/KEAP1 complex. Upon activation, following its dissociation from the KEAP1 complex, Nrf2 undergoes nuclear translocation and initiates transcriptional activation of cytoprotective genes critical for cellular antioxidant defenses and inflammatory regulation. Figure 2D presents a schematic of the experimental cell treatment. Immunofluorescence and Western blotting were utilized to quantify expression of core regulatory proteins critical for the cellular antioxidant system. Under oxidative stress conditions, L-Arg induced nuclear translocation of Nrf2 (Figure 2E–H), suggesting enhanced cellular antioxidant defenses via Nrf2 pathway activation. Western blot analysis demonstrated significantly reduced nuclear Nrf2 expression following H_2_O_2_ incubation, which was markedly reversed by treatment with L-Arg (Figure 2I and J). Our findings demonstrate enhanced Nrf2 nuclear translocation by L-Arg under oxidative stress, with concomitant upregulation of downstream antioxidant gene expression. We observed an inverse correlation between KEAP1 protein and Nrf2 expression levels, and these findings support the hypothesis that L-Arg-mediated Nrf2 pathway activation upregulates antioxidant enzyme expression, thereby mitigating oxidative stress. MDA levels, reflecting cumulative lipid peroxidation from oxidative stress, compromise cellular membrane integrity and diminish endogenous antioxidant defenses [30]. The enzymes SOD and GSH-Px play crucial roles in decomposing peroxides in the body, effectively eliminating oxygen free radicals; maintaining cellular redox balance and reducing oxidative damage to cells, tissues and organs [30, 31]. Antioxidant enzyme activities (SOD, CAT, GSH-Px) and MDA levels were quantified. L-Arg administration significantly enhanced enzymatic antioxidant capacity while suppressing H_2_O_2_-induced intracellular MDA accumulation (Figure 2K)_._

### L-Arg maintains mitochondrial homeostasis in RAW 264.7 cells by upregulating peroxiredoxin 1 expression

**Figure 3.**
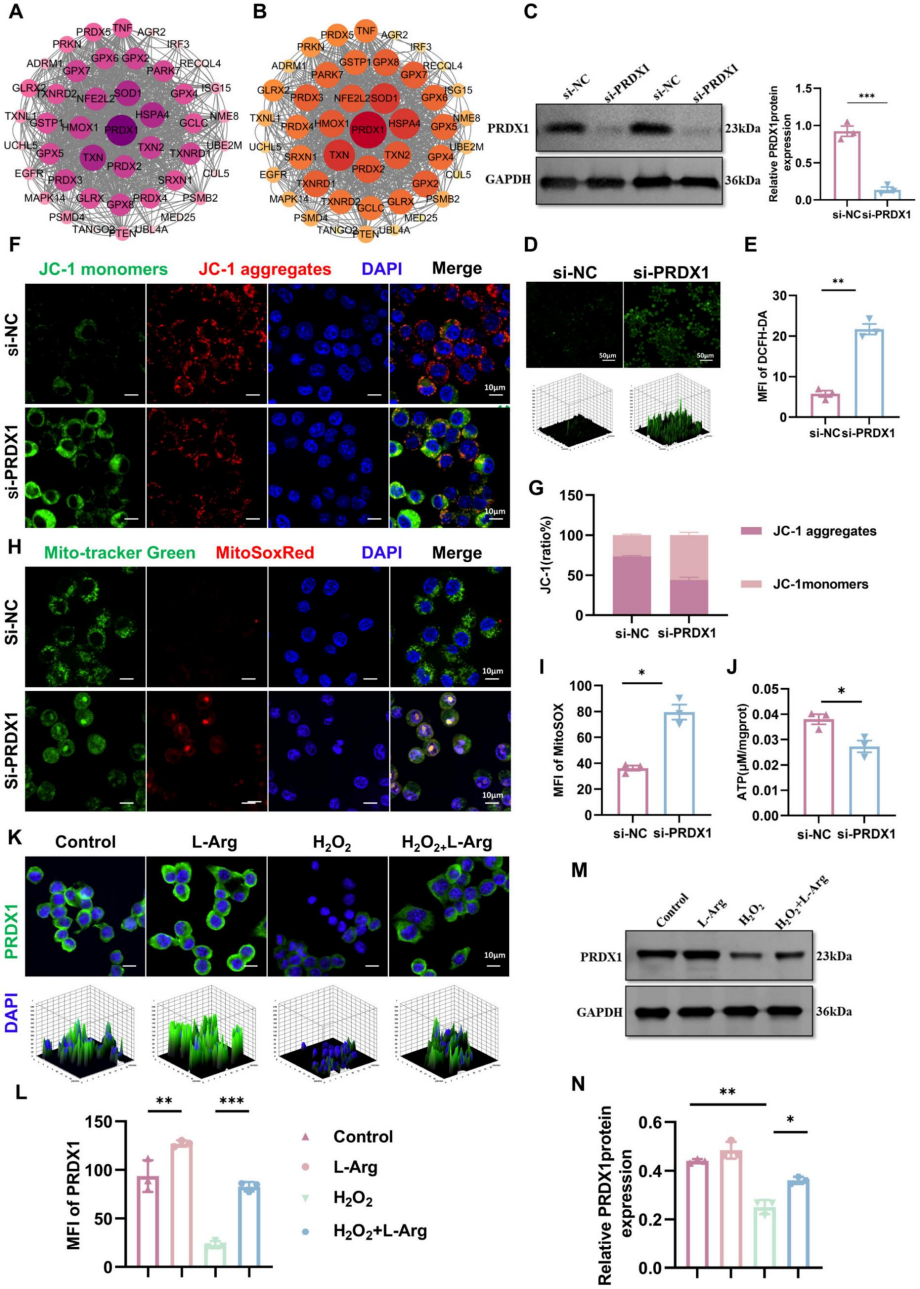
L-Arg upregulates PRDX1 expression and alleviates oxidative stress. (**A, B**) STRING database analysis of PRDX1 PPI network and oxidative damage pathway associations. (**C**) PRDX1 knockdown validation by WB (si-PRDX1, si-NC). (**D, E**) Intracellular ROS levels. (**F, G**) JC-1 fluorescence images and quantitative analysis showing MMP changes. (**H, I**) Mitochondrial superoxide production visualized by MitoSOX Red staining and quantified. (**J**) Quantitative analysis of ATP levels treated with si-NC and si-PRDX1. (**K, L**) PRDX1 immunofluorescence in H_2_O_2_/L-Arg-treated cells. (**M, N**) PRDX1 protein expression by WB. Results are shown as mean ± SD from a minimum of three independent experiments. Statistical comparisons were performed using one-way ANOVA or Student’s *t*-test (*P < 0.05, **P < 0.01, ***P < 0.001).

PRDX1 has been established as essential for maintaining mitochondrial integrity and regulating cellular redox homeostasis [32]. We searched for information on the PRDX1 gene using the GeneCards database and identified its involvement in signaling pathways associated with chemical-induced damage. Using the STRING database, we explored the interaction network of the PRDX1 gene and conducted a visual analysis using Cytoscape (Figure 3A) to illustrate protein–protein interactions (PPIs). The analysis revealed interactions between PRDX1 and adjacent proteins, such as SOD1 and HMOX1, indicating their collaborative functionality. Furthermore, using the PathCards database, we identified genes associated with the oxidative damage response pathway, including PRDX1, for PPI analysis. As shown in Figure 3B, PRDX1 exhibited a hub role within the network. We then employed siRNA technology to knock down PRDX1 (Figure 3C). Relative to si-NC transfection, PRDX1 knockdown caused a notable increase in ROS levels (Figure 3D and E). In cells with normally functioning mitochondria, characterized by a high MMP, the JC-1 dye concentrates in the mitochondrial matrix, generating red fluorescent aggregates, also referred to as JC-1 aggregates. The disruption of MMP hinders JC-1 dye accumulation, resulting in its presence as monomers that emit green fluorescence (JC-1 monomers) [33]. Figure 3F and G shows PRDX1 knockdown markedly decreased JC-1 aggregate fluorescence intensity while simultaneously increasing JC-1 monomer fluorescence intensity, demonstrating impaired MMP. PRDX1 knockdown significantly enhanced mitochondrial superoxide fluorescence intensity as visualized in Figure 3H and I, indicating ROS accumulation. Excessive ROS leads to mitochondrial dysfunction. Mitochondria are central to energy metabolism, we measured ATP production (Figure 3J). The experimental results demonstrated a pronounced decrease in mitochondrial energy generation following PRDX1 knockdown. These results further corroborate the critical role of PRDX1 in preserving mitochondrial homeostasis. Exposure to H_2_O_2_ for 24 h resulted in a pronounced decline in PRDX1 expression. This effect was partially reversed by L-Arg co-treatment (Figure 3K–N). Immunofluorescence imaging technology was utilized to observe the co-localization of PRDX1 and the mitochondrial protein TOM20 within cells. As depicted in Supplementary Figure S1, it was observed that after L-Arg treatment, the expression of PRDX1 increased and its localization with TOM20 was more obvious. These findings indicate that L-Arg enhances cellular defence against oxidative stress by preserving PRDX1 protein expression, thereby alleviating H_2_O_2_-induced oxidative damage and maintaining the cellular redox balance.

**Figure 4.**
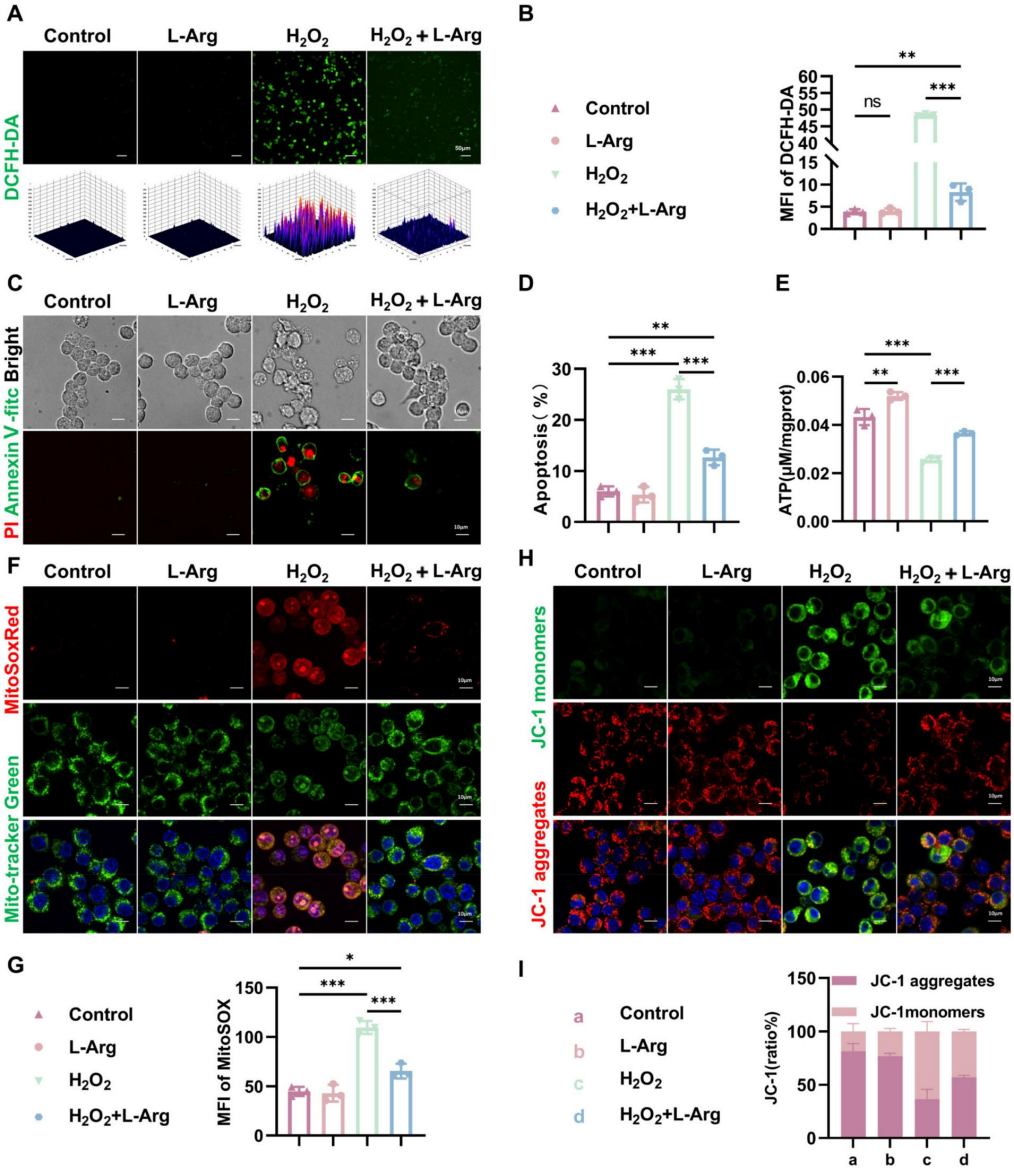
Oxidative stress responses in RAW264.7 macrophages. (**A, B**) Intracellular ROS detection using DCFH-DA fluorescence. (**C, D**) Apoptosis analysis. (**E**) ATP quantification. (**F, G**) MitoSOX Red staining mitochondrial superoxide. (**H, I**) MMP alterations evaluated by JC-1 assay. Data are expressed as mean ± SD from at least three independent experiments. Statistical analyses were conducted using one-way ANOVA (*P < 0.05, **P < 0.01, ***P < 0.001).

Mitochondria are the main site of ROS production. Persistent high ROS lead to mitochondrial dysfunction and increased apoptosis [34]. As shown in Figure 4A–D, our study revealed that L-Arg supplementation significantly reduced H_2_O_2_-induced ROS production and apoptosis. Mitochondria are central to energy metabolism; once damaged, ATP production declines. ATP serves as a key indicator of cell energy metabolism, with increased levels reflecting enhanced cell metabolic activity. Our study examined L-Arg's impact on mitochondrial bioenergetics, demonstrating its ability to significantly enhance ATP generation (Figure 4E). Mitochondrial ROS serve as reliable indicators of both organelle dysfunction and cellular viability. In our experimental design, cells received L-Arg pretreatment (0.5 mM, 2 h) before H_2_O_2_ exposure (200 μM, 24 h). Notably, L-Arg treatment effectively reduced H_2_O_2_-mediated mitochondrial superoxide accumulation (Figure 4F and G). L-Arg counteracted the H_2_O_2_-induced decrease in membrane potential (Figure 4H and I). In conclusion, L-Arg effectively reduced mitochondrial superoxide levels, restored membrane potential, sustained mitochondrial homeostasis and enhanced ATP production by upregulating PRDX1 expression.

### L-Arg promotes M2-type macrophage polarization by regulating PRDX1-mediated mitochondrial metabolic pathways

**Figure 5.**
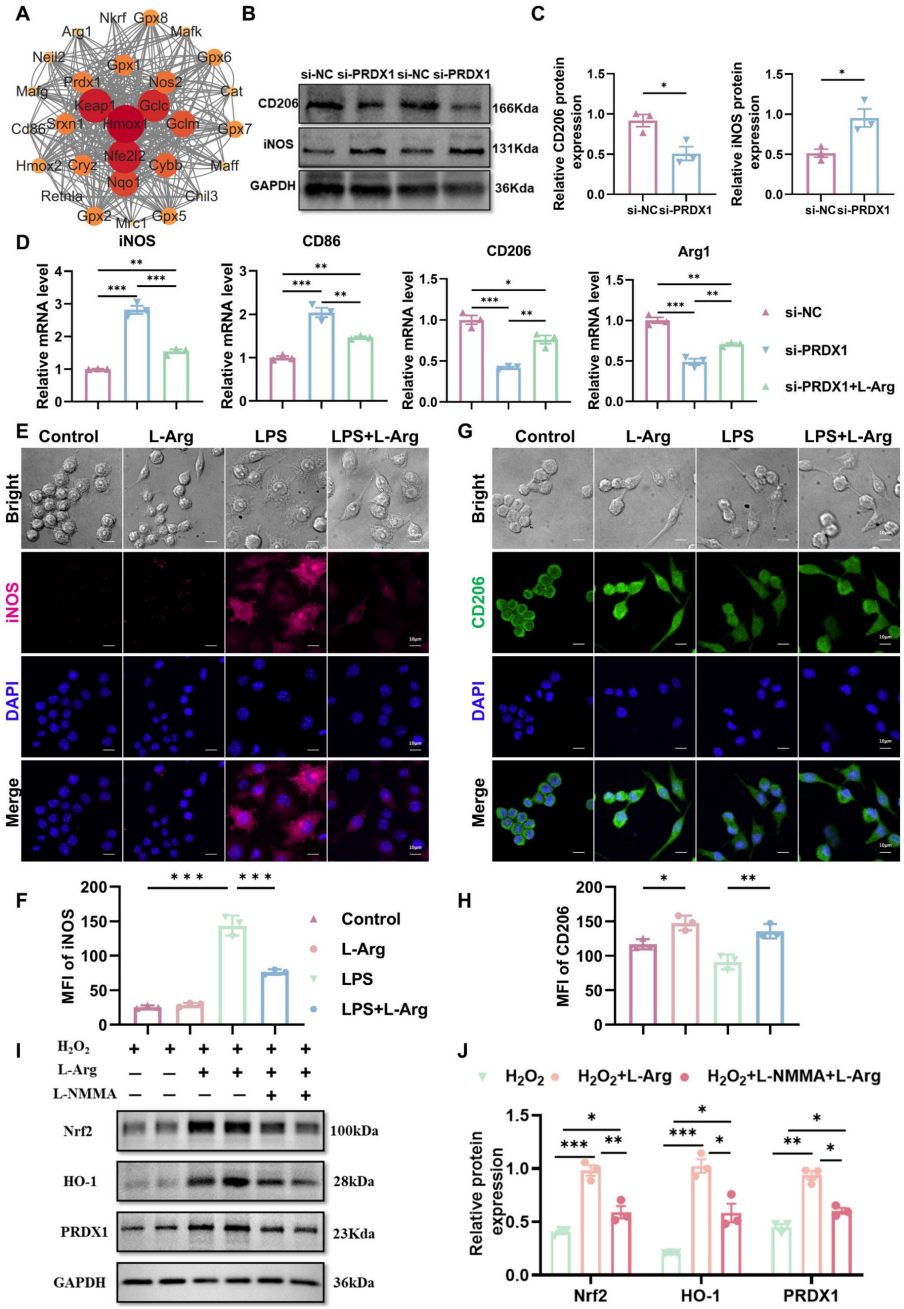
L-Arg modulates macrophage polarization via PRDX1. (**A**) STRING network analysis of PRDX1 interactions with KEAP1–Nrf2–HO-1 and polarization markers. (**B, C**) Western blot of CD206, iNOS in si-PRDX1 cells. (**D**) qRT-PCR of polarization markers with different treatments. (**E–H**) Immunofluorescence visualization and quantification of iNOS, CD206 in L-Arg/LPS-treated cells. (**I, J**) Western blot analysis of Nrf2, HO-1 and PRDX1 expression in cells under different treatments. Data are expressed as mean ± SD from at least three independent experiments. Statistical analyses were conducted using one-way ANOVA or Student’s *t*-test (*P < 0.05, **P < 0.01, ***P < 0.001).

We conducted a detailed analysis of the classical oxidative stress pathway of KEAP1–Nrf2–HO-1, as well as PRDX1, and the STRING interaction network among macrophage phenotypes CD206, iNOS, CD86 and Arg1 (Figure 5A), which further highlights their interrelationship. Furthermore, our findings provide compelling evidence that PRDX1 knockdown induces a shift toward pro-inflammatory polarization, favoring the M1 phenotype, while inhibiting reparative polarization associated with the M2 phenotype in macrophages (Figure 5B and C). Figure 5D further supports the important role of L-Arg in regulating macrophage polarization through PRDX1. The metabolism of L-Arg is considered a crucial metabolic regulatory factor that modulates macrophage polarization and inflammatory responses [9]. Macrophages were exposed to 0.5 mM L-Arg and 200 ng/ml LPS for 24 h to evaluate the impact of L-Arg on macrophage function. Immunofluorescence staining was performed (Figure 5E–H). White light microscopy showed M1 macrophages displaying typical ‘fried egg’ morphology, contrasting with the elongated, rod-shaped M2 phenotype. Quantitative analysis revealed that L-Arg treatment significantly reduced M1 marker iNOS expression compared to the LPS controls, while enhancing M2-associated CD206 levels. ELISA revealed significant reductions in TNF-α and IL-1β levels and a significant increase in IL-10 level in the supernatant of L-Arg-treated cells (Supplementary Figure S2). These results align with the inhibition of M1 macrophage activity and the promotion of M2 macrophage activation, confirming that L-Arg reprograms macrophage polarization from the M1 to the M2 phenotype. L-Arg serves as the sole obligate substrate for nitric oxide (NO) generation. To dissect the contribution of downstream NO signaling, we pharmacologically suppressed NO synthesis with the pan-NOS inhibitor L-NMMA (0.5 mM) (Figure 5I and J). This intervention attenuated PRDX1 expression and impaired activation of the KEAP1–Nrf2–HO-1/PRDX1 antioxidant axis. Collectively, these findings demonstrate NOS-derived NO modulates mitochondrial metabolic reprogramming via the KEAP1–Nrf2–HO-1/PRDX1 pathway, thereby inhibiting M1 polarization and facilitating macrophage commitment to the M2 phenotype.

### Characterization of the L-Arg-loaded microneedle

**Figure 6.**
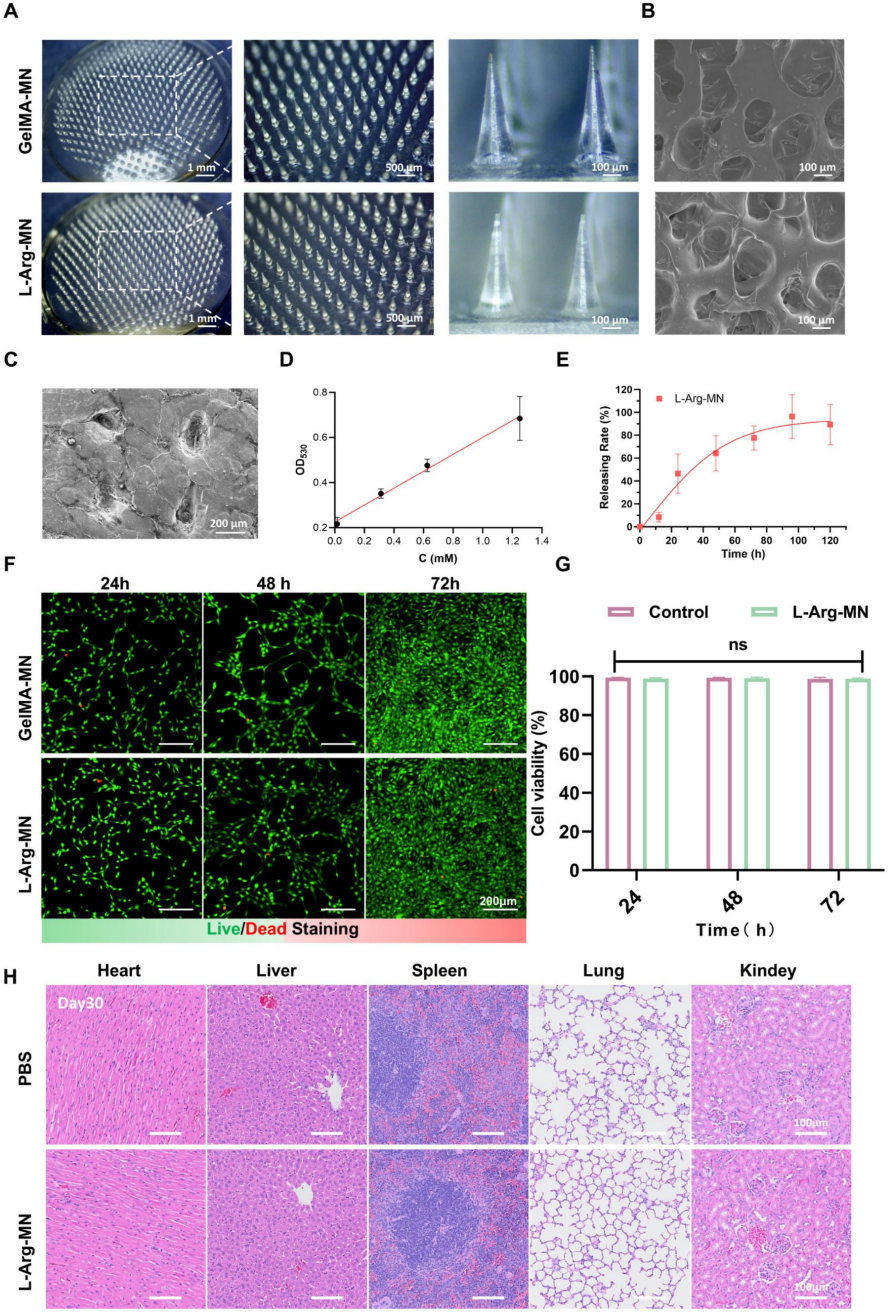
Characterization of L-Arg-MN. (**A**) Optical images of GelMA-MN vs L-Arg-MN. (**B**) SEM showing porous microstructure. (**C**) Skin penetration capability. (**D**) L-Arg quantification standard curve. (**E**) *In vitro* L-Arg release profile. (**F, G**) Biocompatibility assessment (cell viability). (**H**) H&E-stained major organs after 30-day treatment. Results are shown as mean ± SD from a minimum of three independent experiments. Statistical comparisons were performed using one-way ANOVA (*P < 0.05, **P < 0.01, ***P < 0.001, ns = not significant).

L-Arg exhibits significant antioxidant properties that effectively regulate inflammation. However, its local administration at wound sites is hindered by challenges such as poor retention, hyperosmolar tissue damage and limited penetration into deeper tissues, all of which restrict its clinical applicability. To achieve sustained and effective delivery of L-Arg to the wound, GelMA was employed as a carrier. L-Arg was evenly mixed with a GelMA and LAP solution, cast into a mold and UV-cured to produce L-Arg-MN. Figure 6A illustrates the morphology of both GelMA-MN and L-Arg-MN, showing a well-ordered array with uniform tips and bases. This design ensures minimal pain and reduces tissue damage during the penetration process. SEM images (Figure 6B) revealed that the hydrogels possessed interconnected porous structures. Figure 6C demonstrates successful MN penetration through the stratum corneum, with clearly visible needle-shaped formations in the dermal layers. This observation confirms their strong penetration ability, facilitating the effective delivery of L-Arg to deeper skin layers, thereby enhancing drug absorption efficiency and reducing surface retention. However, L-Arg—being an alkaline amino acid—can markedly elevate local pH at supraphysiological concentrations (Supplementary Figure S3), potentially perturbing the wound microenvironment and amplifying the inflammatory response. The biodegradability of GelMA enables the controlled and gradual release of L-Arg, improving drug stability and avoiding abrupt drug release. The *in vitro* release kinetics of L-Arg were quantified spectrophotometrically at 530 nm (Figure 6D). As shown in Figure 6E, L-Arg exhibited time-dependent release from the MN system, with an initial progressive increase followed by stabilization, demonstrating sustained delivery capability. Continuous low-dose delivery sustains the pro-healing efficacy of L-Arg while preventing pH-mediated inflammatory exacerbation that can arise from transient supraphysiological concentrations. Figure 6F and G shows the impact of GelMA-MN and L-Arg-MN on cell viability at 24, 48 and 72 h. L-Arg-MN has no significant influence on cell viability. As shown in Supplementary Figure S4, the degradation of L-Arg-MN was evaluated using H&E staining of tissue sections. The histological analysis revealed that the basic degradation of microneedles could be observed around the 10th day. Supplementary Figure S5 and Figure 6H, within the diabetic mouse model, we evaluated the effects of L-Arg-MN on the heart, liver, pancreas, spleen and kidneys through histopathological analysis. No apparent inflammation, necrosis or organ damage was seen. L-Arg-MN did not cause significant pathological changes in major organs during prolonged observation. These findings indicate that L-Arg-MN possesses good biocompatibility. In summary, L-Arg-MN, as an innovative MN-based delivery system, exhibits a remarkable drug-loading capacity, sustained-release performance and favorable biocompatibility.

### L-Arg-loaded microneedle promotes diabetic wound healing

Diabetic wound healing is largely impeded by persistent oxidative stress and a prolonged inflammatory response [35]. To determine if L-Arg-MN can accelerate diabetic wound healing *in vivo*, we generated a full-thickness skin defect model in diabetic mice. The wounds were photographed, sampled and stained at various time points (Figure 7A). A series of experimental studies was subsequently conducted. L-Arg-MN treatment significantly enhanced wound closure rates in diabetic mice relative to the untreated controls (Figure 7B–D), demonstrating its therapeutic potential for impaired diabetic wound healing. Wound tissues from the three groups were harvested for H&E, Masson’s and immunofluorescence staining to examine neo-granulation tissue formation, collagen deposition and tissue-specific immunofluorescence. The L-Arg-MN treatment significantly enhanced granulation tissue regeneration and collagen deposition (Figure 7E–H). Additionally, the *in vivo* experiment results aligned with the *in vitro* observations. As depicted in Figure 7I and J, L-Arg-MN significantly increased Nrf2, HO-1 and PRDX1 expression levels relative to the control group, while KEAP1 expression decreased. Supplementary Figure S6, which shows the measurements of antioxidant enzymes (SOD, CAT, GSH) and MDA levels on days 5 and 7, further confirmed L-Arg's antioxidant effect and its role in reducing oxidative stress in wounds by activating the KEAP1–Nrf2–HO-1 pathway and upregulating PRDX1. ROS levels were evaluated by staining frozen sections with DHE. L-Arg and L-Arg-MN treatments substantially decreased ROS levels relative to the controls (Figure 7K and L), demonstrating their antioxidant capacity to counteract oxidative stress in diabetic wounds. As shown in Supplementary Figure S7, on the 7th day of the wound surface, the tissue ROS level further decreased, facilitating the transition of the wound from the inflammatory to the proliferative phase. Additionally, Figure 7M–T shows that in wounds treated with L-Arg and L-Arg-MN, the expression of PRDX1 and Ki67, as well as M2 macrophage polarization increased, while M1 macrophages decreased. This shift promoted tissue proliferation and indicated that L-Arg can effectively mitigate wound inflammation and promote immune homeostasis during the repair process. It was observed that this promoting effect was more obvious in the tissue sections on the 10th day of the wound, as depicted in Supplementary Figures S8–S10, demonstrating the sustained effect of L-Arg-MN and its potential to promote wound repair. Furthermore, ELISA analysis of the wound tissue on days 7 and 10 revealed that L-Arg-MN treatment significantly reduced the levels of pro-inflammatory cytokines TNF-α and IL-1β, while increasing the level of anti-inflammatory cytokine IL-10 (Supplementary Figure S11). These results further support the notion that L-Arg-MN promote immune homeostasis during the repair process. On day 10 following treatment, vascular CD31 expression was notably elevated in the L-Arg treatment group, underscoring its advantageous effect in enhancing angiogenesis in diabetic wounds (Figure 7U and V). Our results indicate that L-Arg, through its antioxidant and immunomodulatory effects, promotes wound progression from inflammation to proliferation, thereby enhancing tissue repair.

**Figure 7.**
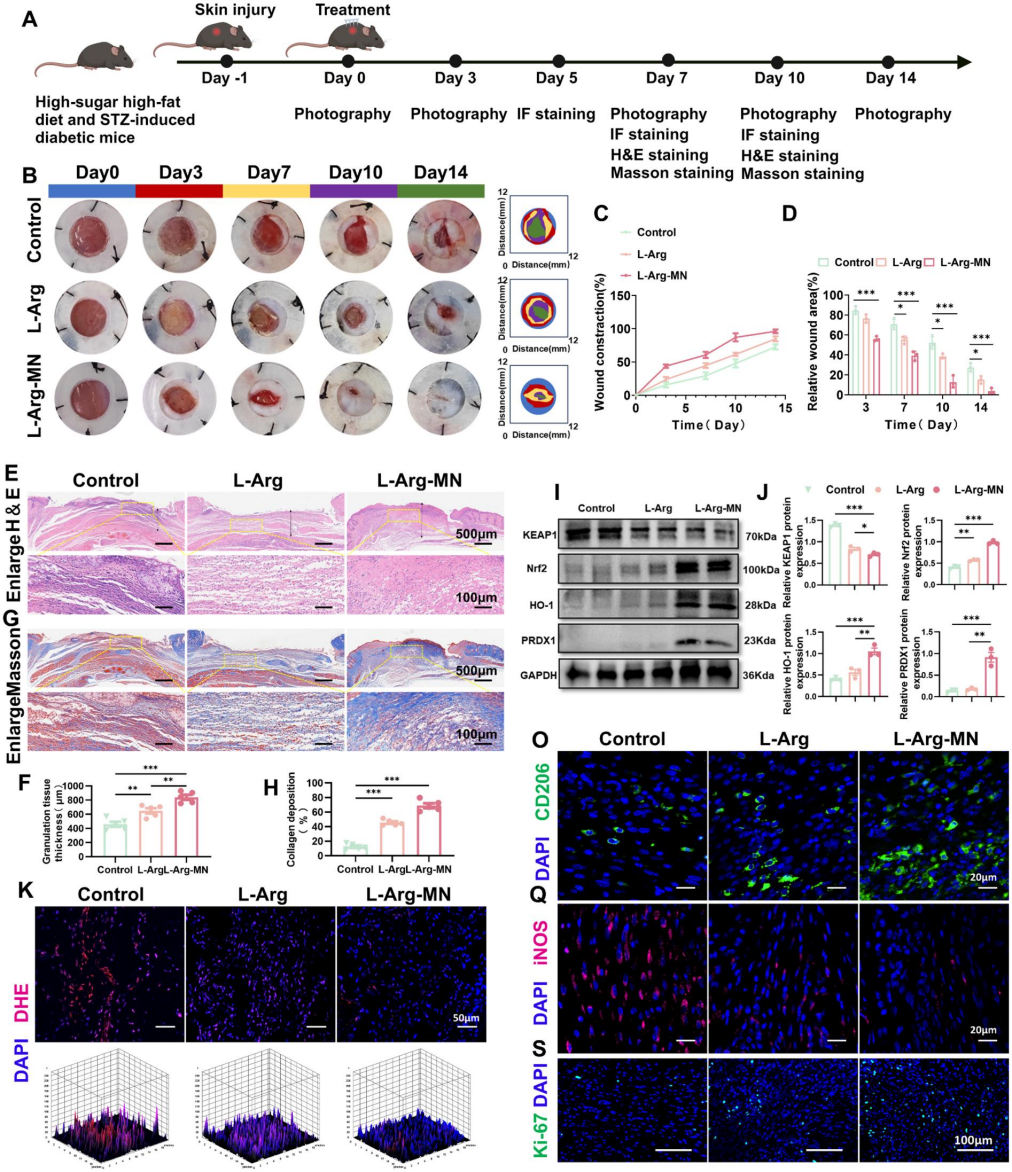
L-Arg-MN promotes diabetic wound healing. (**A**) Experimental schematic. (**B**) Wound appearance at indicated timepoints. (**C, D**) Quantification of healing rates and wound areas. (**E–H**) Histology (H&E and Masson's) on day 7. (**I, J**) WB of KEAP1/Nrf2/HO-1/PRDX1. (**K, L**) DHE staining (day 5) and ROS quantification. (**M, N**) PRDX1 immunofluorescence (day 7). (**O–T**) Immunofluorescence of CD206/iNOS/Ki67 (day 7). (**U, V**) CD31 staining (day 10). Results are shown as mean ± SD from a minimum of three independent experiments. Statistical comparisons were performed using one-way ANOVA (*P < 0.05, **P < 0.01, ***P < 0.001).

**Figure 7.**
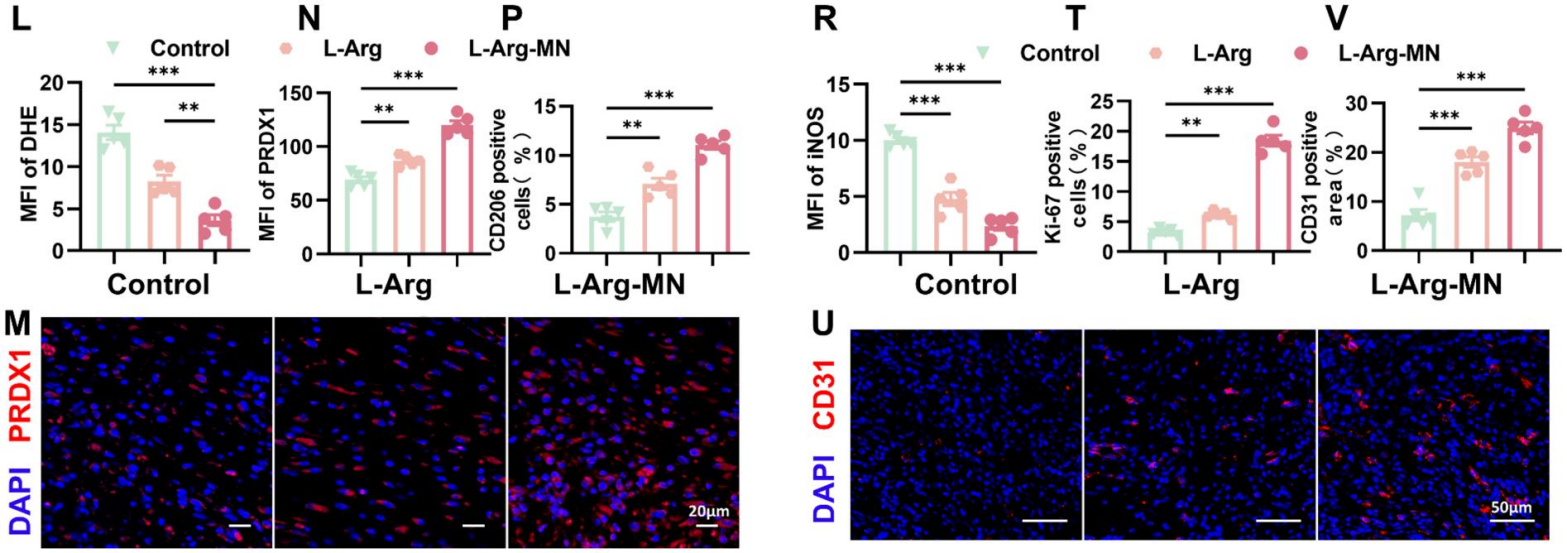
(Continued).

## Discussion

Diabetic wound healing is a complicated and precisely managed sequence of events, including phases such as haemostasis, inflammation, proliferation and tissue remodeling [36]. The effective advancement of these stages is significantly hampered by chronic inflammation and oxidative stress, which frequently results in poor wound healing in diabetics [37, 38]. Our study demonstrates that L-Arg-loaded MN enhance diabetic wound healing by modulating macrophage polarization and mitochondrial homeostasis.

L-Arg effectively mitigates oxidative stress by regulating key antioxidant enzymes and preserving mitochondrial integrity. Specifically, we observed upregulation of SOD, CAT and GSH-Px activities and decreased MDA levels. These findings are consistent with earlier studies demonstrating that L-Arg activates the KEAP1–Nrf2–HO-1 pathway, a key pathway of cellular antioxidant defense [39, 40]. Nrf2 activation and subsequent nuclear translocation are essential for inducing antioxidant gene expression, thereby protecting various tissues, particularly healing wounds, from oxidative damage [41]. Furthermore, our study builds upon previous findings by showing that L-Arg upregulates PRDX1, a key protein involved in maintaining mitochondrial redox balance [42]. PRDX1 plays a critical role in scavenging mitochondrial superoxide radicals, restoring MMP and enhancing ATP production [43], all of which contribute to improved cellular function during wound healing. This aligns with prior research by Song *et al.*, who reported that PRDX1 helps prevent mitochondrial dysfunction by inhibiting ROS-induced damage, which can lead to ferroptosis in colorectal cancer cells [44]. These findings indicate that PRDX1 protects mitochondrial function under stress conditions [45]. Our findings offer strong proof that PRDX1 is essential for the positive effects of L-Arg on antioxidant defense and mitochondrial homeostasis. Macrophage polarization—a dynamic process that significantly influences inflammation and tissue repair—is another key factor in wound healing [46]. Huang *et al.* demonstrated that sulforaphane and Ding *et al.* reported that resveratrol both accelerate M2 macrophage polarization and enhance diabetic wound closure [47, 48]. L-Arg promotes macrophage polarization from M1 to M2 phenotypes through PRDX1-dependent mitochondrial regulation. As M2 macrophages are acknowledged for their anti-inflammatory characteristics and function in tissue repair, this phenotypic transition is vital for decreasing inflammation and advancing tissue regeneration. Our results are in line with those of Zhu *et al*. [49], who found that PRDX1 inhibits the MAPK pathway to lower apoptosis in myocardial ischaemia/reperfusion damage, with ROS acting as an upstream regulator of this apoptotic process. Anti-inflammatory M2 phenotypic shift from pro-inflammatory M1 phenotype is typically linked to inhibition of the MAPK pathway [50]. It has been demonstrated that L-Arg alters the polarization of macrophages during wound healing, enhancing M2 macrophage infiltration and promoting tissue regeneration in diabetic mice [9]. Our study results further confirm the essential contribution of M2-type macrophages to wound healing. In wounds treated with L-Arg-MN, we observed higher collagen deposition, improved granulation tissue development and quicker wound closure. L-Arg orchestrates macrophage repolarization from the pro-inflammatory M1 to the resolving M2 phenotype through a multimodal program. PRDX1-dependent mitochondrial remodeling functions as the dominant driver, while arginase-catalyzed ornithine flux and chromatin acetylation provide complementary layers of control. The precise circuitry by which L-Arg engages the KEAP1–Nrf2–HO-1/PRDX1 axis remains unresolved. Pharmacological blockade with the pan-NOS inhibitor L-NMMA attenuated, yet did not abolish, activation of this axis, indicating that NO generated via the NOS route is only one of several metabolites or signals sustaining the transcriptional cascade [51]. NO can modify KEAP1 via cysteine S-nitrosation or S-guanylation, disrupting KEAP1–Nrf2 binding and enabling Nrf2 nuclear translocation to transcribe antioxidant genes like HO-1 and PRDX1 [52–54]. Potential Nrf2–PRDX1 positive feedback or contributions from other metabolites require further investigation [55–57]. Notably, this study establishes L-Arg's functional activation of this axis and its enhancement of antioxidant capacity, underpinning the therapeutic efficacy of our microneedle platform. Future conditional knockout models, isotope tracing and single-cell multi-omics will dissect the relative contributions and refine dose–efficacy–toxicity relationships across species.

Microneedles offer painless, minimally invasive, highly efficient and multifunctional therapeutic modalities for diabetic wounds [58], yet their clinical implementation remains constrained by restricted drug-loading capacity and penetration depth, suboptimal mechanical robustness, considerable inter-individual skin variability, a paucity of regulatory standards and the imperative for additional translational validation. The development of the L-Arg-MN delivery system represents a notable advancement in the treatment of diabetic wounds. Conventional drug delivery methods are often limited by rapid clearance and poor retention at the wound site. Conversely, the GelMA-MN system offers sustained and targeted delivery of L-Arg to deeper tissue layers, reducing local irritation and enhancing therapeutic efficacy. This sustained-release system overcomes conventional delivery limitations by prolonging L-Arg retention at wound sites, significantly improving therapeutic efficacy. These results corroborate Liu *et al.*'s [59] demonstration of microneedle-enhanced localized drug delivery for chronic wound treatment. Although the L-Arg-MN microneedle patch achieved certain meaningful results in animal models in this study, before applying it to humans, it is still necessary to deeply explore the possible challenges in its clinical transformation process. Skin variability arises from inherent differences between animal and human skin, as human skin exhibits greater heterogeneity in stratum corneum thickness, appendage distribution and immune responses, which may affect microneedle penetration efficiency and drug absorption [60, 61]. Drug delivery stability, while preclinical studies demonstrate controlled L-Arg release, clinical conditions, varying skin hydration, mechanical stress and storage environments could alter release kinetics and drug stability [62]. Manufacturing scalability, ensuring batch-to-batch consistency in microneedle geometry, drug loading and mechanical strength, remains a challenge for large-scale production, particularly when transitioning from lab-scale to industrial manufacturing [63]. These limitations highlight the need for further optimization and validation before clinical deployment.

While our study presents promising findings, several limitations remain. Although we have shown that L-Arg upregulates PRDX1 expression, the precise molecular mechanisms underlying this regulation warrant further exploration. Additionally, more investigation is required to examine how L-Arg affects macrophage polarization in diverse inflammatory settings as well as the spatiotemporal pharmacokinetic profiles of L-Arg released from the microneedle array within the local wound milieu and the systemic circulation. This study provides a novel strategy for transdermal drug delivery and has achieved meaningful results in animal models. However, the long-term efficacy and safety of L-Arg-MN remain to be confirmed, and human trials have not yet been conducted, as discussed by Wu *et al.* [64]. Future research should focus on long-term clinical trials, expansion of sample sizes, in-depth investigation of drug release mechanisms, optimization of manufacturing processes and exploration of new application areas [65]. These efforts will enhance the study's integrity and scientific rigor and facilitate its clinical translation.

In conclusion, this study identifies new pathways through which L-Arg promotes the healing of diabetic wounds by means of PRDX1-mediated mitochondrial homeostasis and the KEAP1–Nrf2–HO-1 signaling axis. The L-Arg-MN delivery system exhibits potential as a targeted wound therapy, addressing the key limitations of conventional drug delivery methods. These findings indicate the potential clinical application of L-Arg in chronic wound management and provide the basis for developing therapies targeting inflammation-associated diseases.

## Conclusion

To sum up, this study indicates that L-Arg upregulates PRDX1 expression and activates the KEAP1–Nrf2–HO-1 signal transduction pathway to reduce oxidative stress and regulate macrophage polarization to promote the healing of diabetic wounds. Additionally, it highlights the molecular mechanism by which L-Arg affects macrophage polarization via regulating mitochondrial homeostasis through PRDX1. Furthermore, developing a GelMA-based L-Arg-MN delivery system offers a sustained and targeted approach to drug administration. With dual metabolic and immunodulatory functions, this innovative approach effectively maintains mitochondrial homeostasis and alleviates oxidative stress, thereby addressing the key limitations of conventional drug delivery methods in diabetic wound therapy. These findings lay the theoretical groundwork for future immunotherapeutic approaches that target PRDX1 and mitochondrial homeostasis, in addition to offering fresh insights into the process underpinning diabetic wound healing.

## Supplementary Material

rbaf092_Supplementary_Data
