# Supplementary material for: L-arginine-loaded microneedle patch enhances diabetic wound healing by regulating macrophage polarisation and mitochondrial homeostasis
Source: Regen Biomater. 2025 Sep 1;12:rbaf092. doi: 10.1093/rb/rbaf092 (PMC12493038; doi:10.1093/rb/rbaf092)
Supplement: rbaf092_Supplementary_Data [file rbaf092_supplementary_data.zip › Supplementary materials.docx]

**Supplementary materials**

**L-Arginine-Loaded Microneedle Patch Enhances Diabetic Wound Healing by Regulating Macrophage Polarisation and Mitochondrial Homeostasis**

Hong Wang^1,†^, Shun Yao^1,†^, Qingyun Mo^1,2^, Mingyue Chen^3^, Danfeng He^1^, Lingfeng Yan^1^, Chang Wang^1^, Tao Zou^1*^, Gaoxing Luo^1*^, Jun Deng^1*^

1 Institute of Burn Research, State Key Laboratory of Trauma and Chemical Poisoning, the First Affiliated Hospital of Army Medical University (the Third Military Medical University), Chongqing 400038, China;

2 School of Medicine, Southeast University, Nanjing, China

3 College of Bioengineering, Chongqing University, Chongqing, China

† These authors contributed equally to this work

**Materials and methods**

***Detection of inflammatory factors***

The levels of the inflammatory cytokines IL-10, TNF-α, and IL-1β were measured using ELISA kits (LunChangShuoBiotech, Xiamen, China). RAW264.7 cells were subjected to various treatments, and the supernatants were collected. These supernatants were then centrifuged at 1500 rpm for 10 minutes at 4°C to remove cellular debris. The supernatants were analyzed using the ELISA kits, following the manufacturer's protocols.

Tissue samples from the wounds were processed by mixing them with PBS that included protease inhibitors (P1050, Beyotime, China) at a ratio of 1:9 (weight to volume). The samples were then homogenized and centrifuged at 5000 rpm for 10 minutes. The supernatant obtained after centrifugation was used for ELISA analysis.

***pH measurement***

L-Arg was dissolved in deionized water to obtain 0.25–512 mM solutions. pH was measured in triplicate with a calibrated pH meter (Shanghai Leici, China) at room temperature.

**
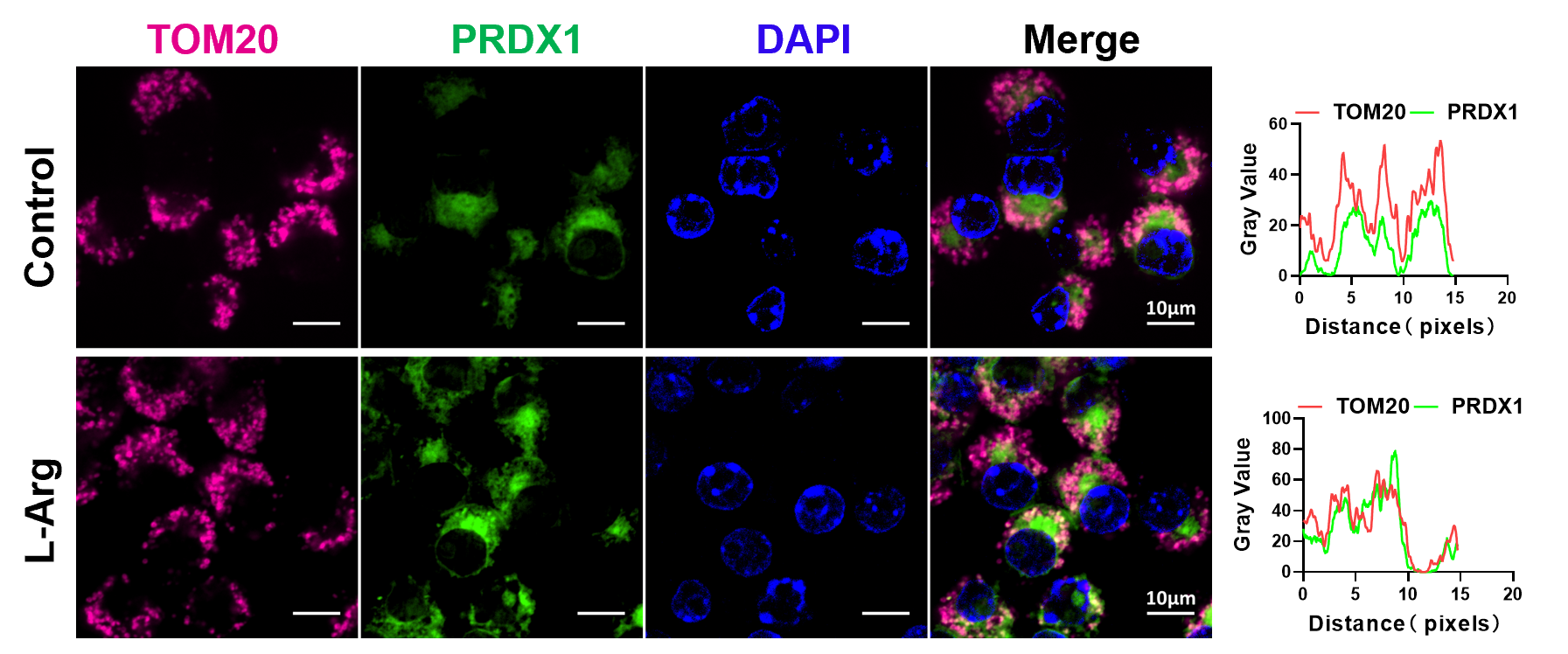
**

**Figure S1.** Immunofluorescence imaging illustrates the co-localization of PRDX1 and TOM20 within cells. The fluorescence intensity profiles further quantify the co-localization of these two proteins and relative expression levels. Scale bars: 10 μm.


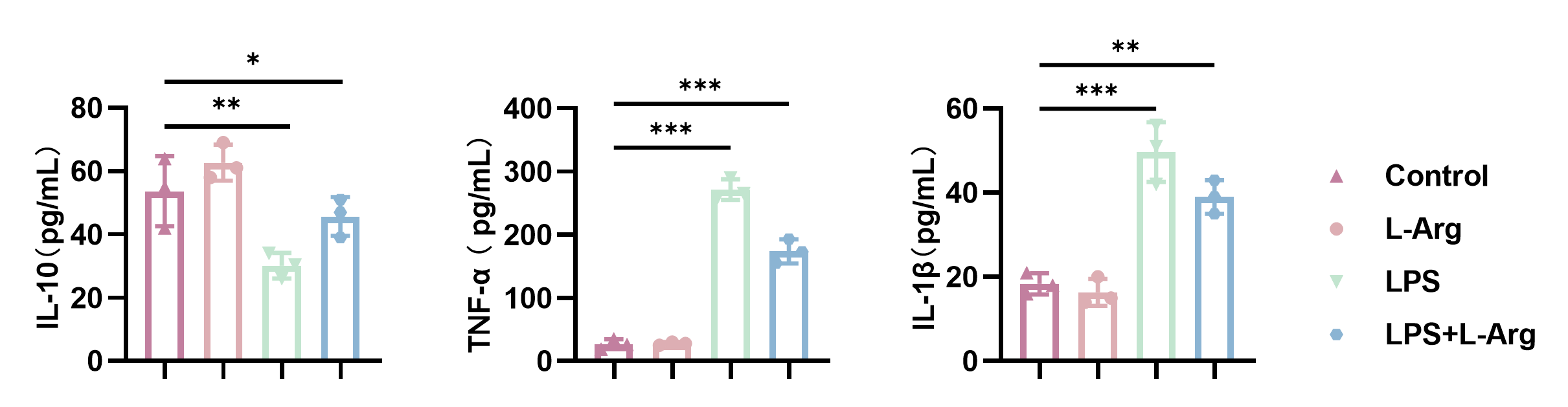


**Figure S2.** IL-10, TNF-α and IL-1β in cell supernatant by ELISA (n =3). Data are expressed as mean ± SD from at least three independent experiments. Statistical analyses were conducted using one-way ANOVA (**P* < 0.05, ***P* < 0.01, ****P* < 0.001).

**Figure S3.** pH-concentration profile of L-Arg dissolved in deionized water at 25°C (n = 3).

**
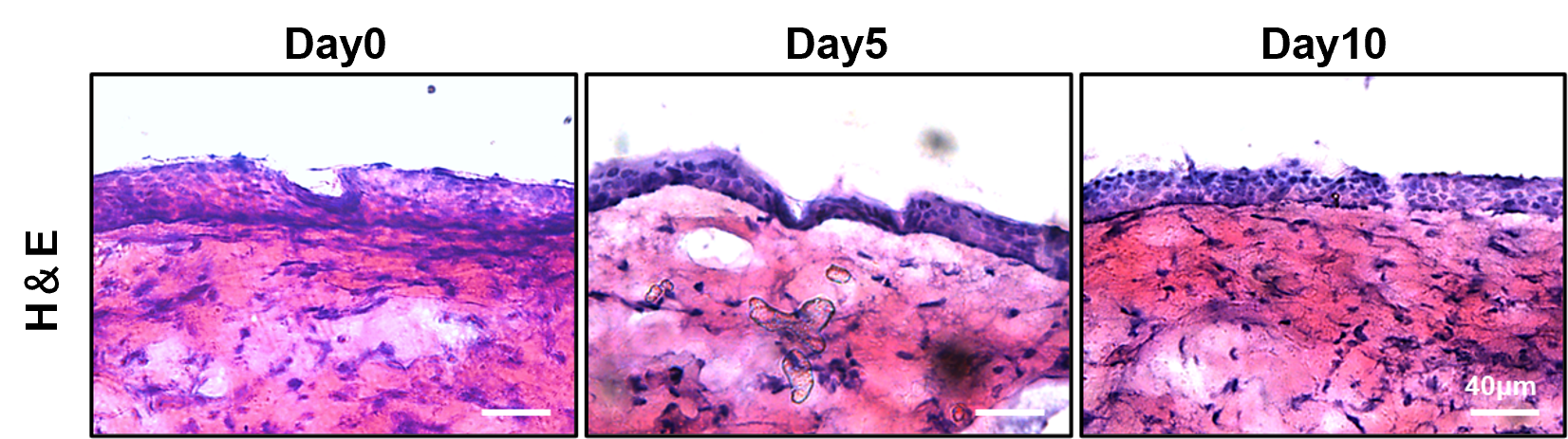
**

**Figure S4.** H&E staining of frozen sections of tissues containing L-Arg-MN at different time points. Scale: 40 μm.


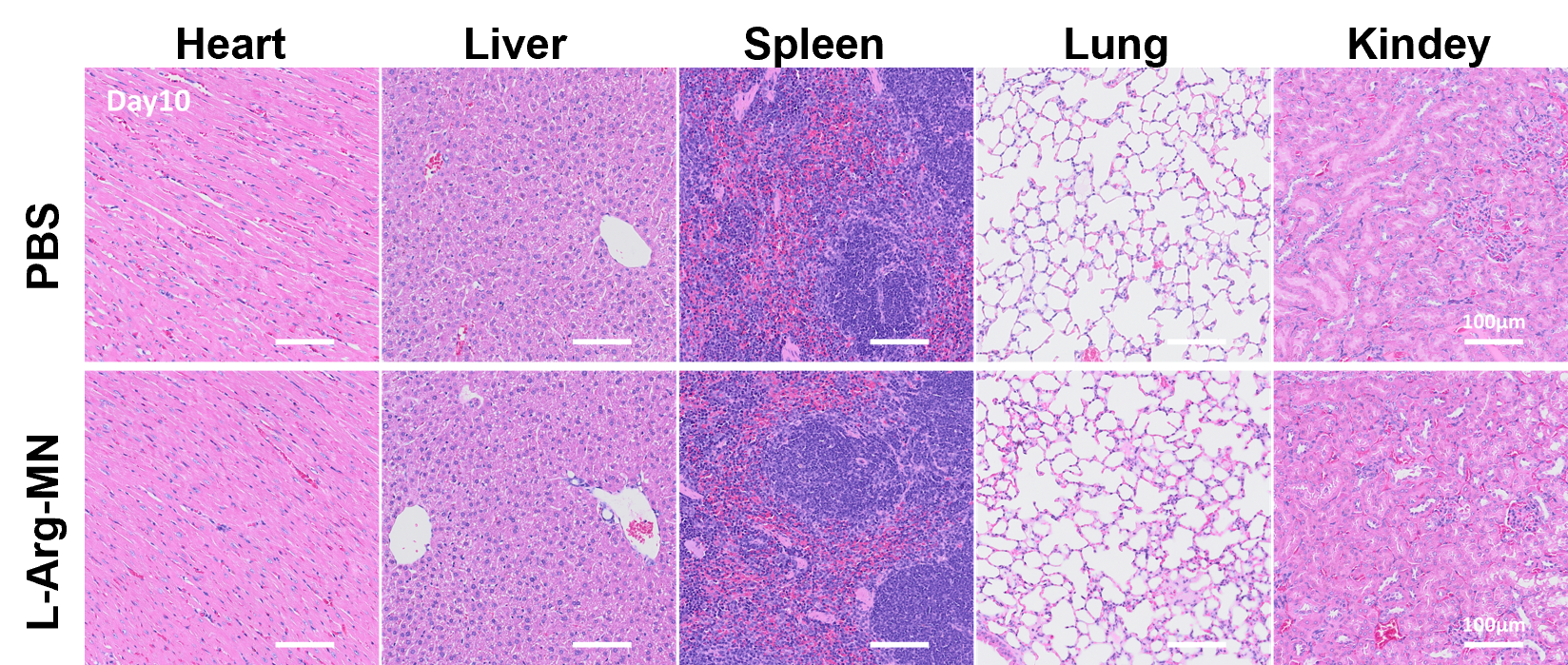


**Figure S5.** H&E staining of typical organs of mice under different treatment conditions for 10 days. Scale bars: 100 µm.

**
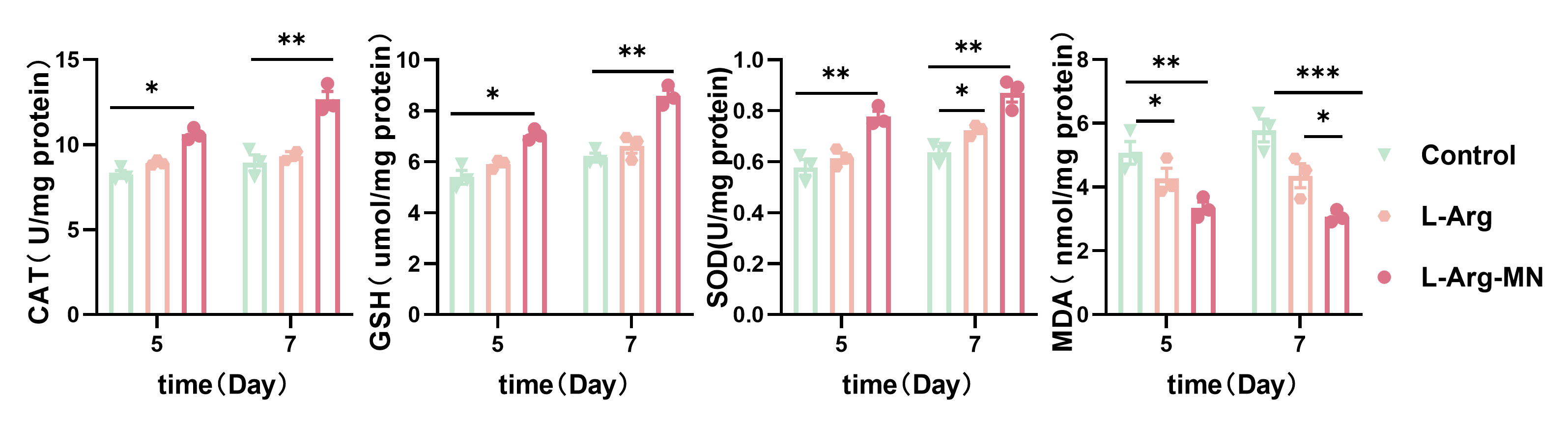
Figure S6.** Enzymatic activity assays for CAT, SOD, GSH-Px and MDA levels in wound tissues on days 5 and 7 (n =3). Results are shown as mean ± SD from a minimum of three independent experiments. Statistical comparisons were performed using one-way ANOVA (**P* < 0.05, ***P* < 0.01, ****P* < 0.001).


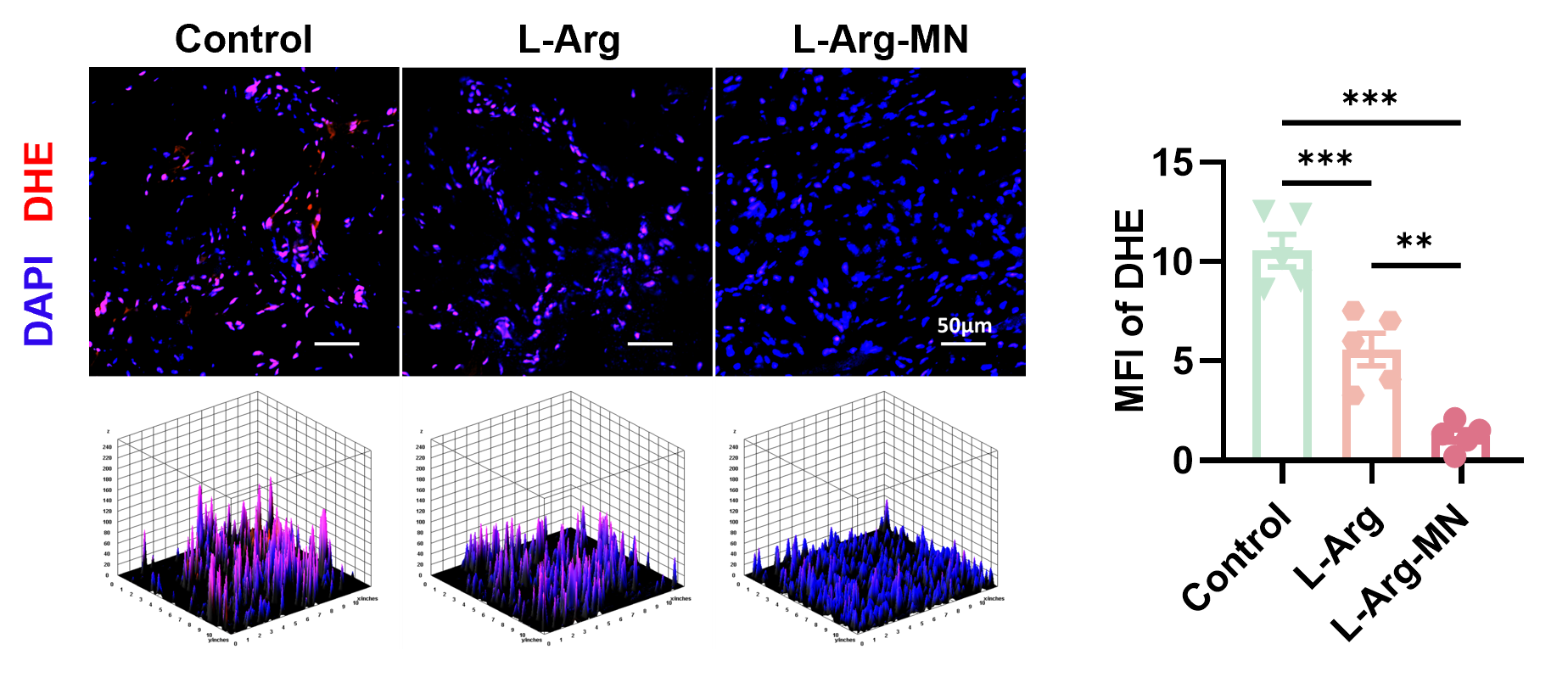
**Figure S7.** DHE staining showing ROS levels in wound tissues on day 7, with corresponding quantitative fluorescence analysis (n = 5). Results are shown as mean ± SD from a minimum of three independent experiments. Statistical comparisons were performed using one-way ANOVA (***P* < 0.01, ****P* < 0.001).

**
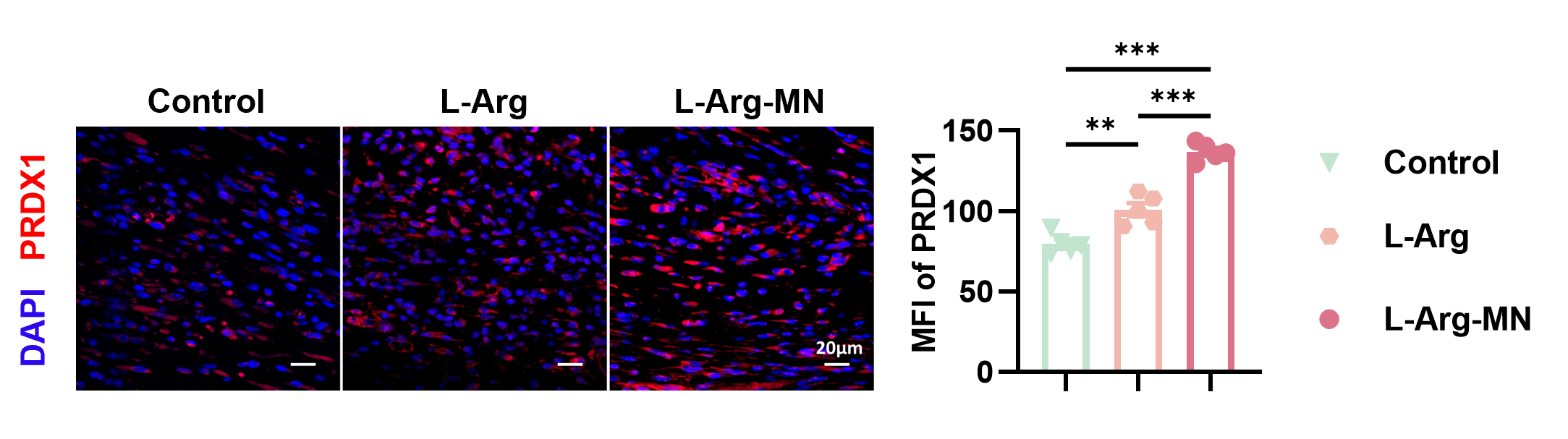
Figure S8.** PRDX1 immunofluorescence staining in wound tissues on day 10 and quantitative fluorescence analysis (n = 5). Results are shown as mean ± SD from a minimum of three independent experiments. Statistical comparisons were performed using one-way ANOVA (***P* < 0.01, ****P* < 0.001).


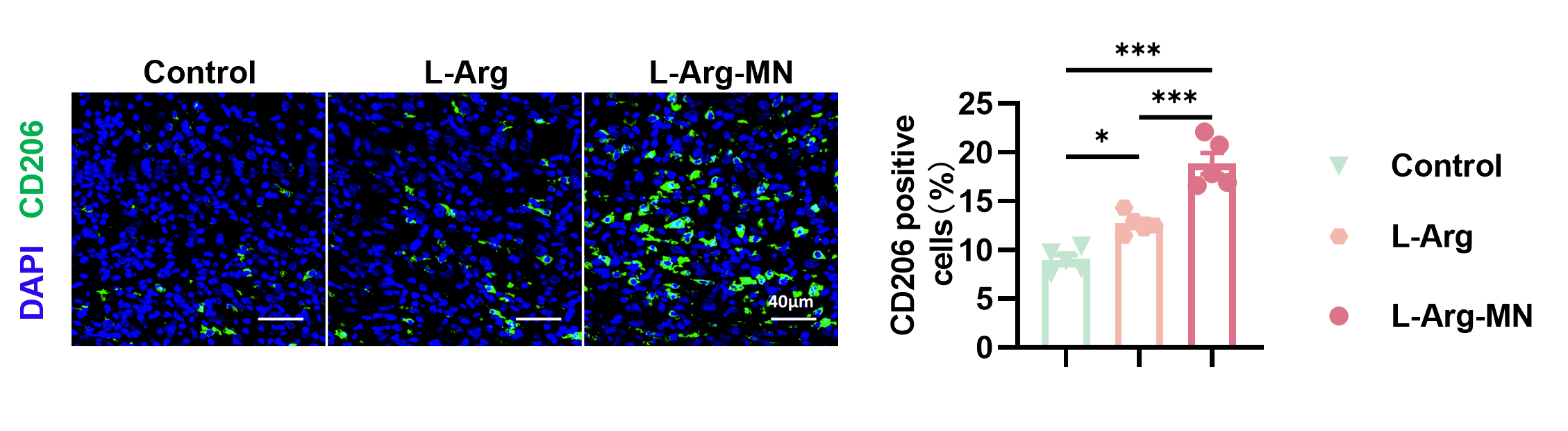
**Figure S9.** Immunofluorescence images showing expression of CD206 in wound tissues on day 10 and quantitative fluorescence analysis (n = 5). Results are shown as mean ± SD from a minimum of three independent experiments. Statistical comparisons were performed using one-way ANOVA (**P* < 0.05, ****P* < 0.001).


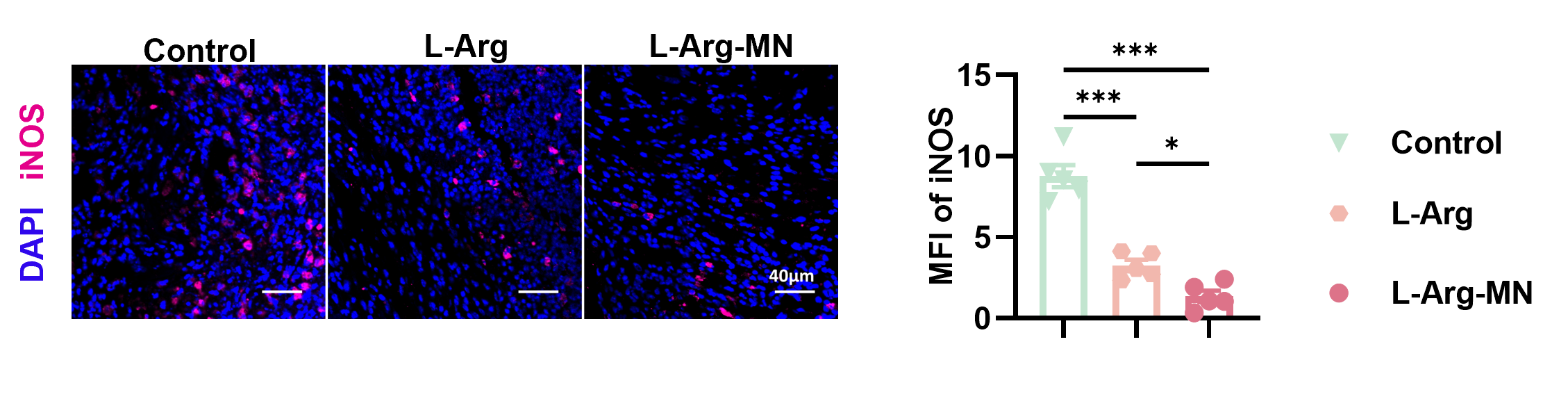


**Figure S10.** Immunofluorescence images showing expression of iNOS in wound tissues on day 10 and quantitative fluorescence analysis (n = 5). Results are shown as mean ± SD from a minimum of three independent experiments. Statistical comparisons were performed using one-way ANOVA (**P* < 0.05, ****P* < 0.001).


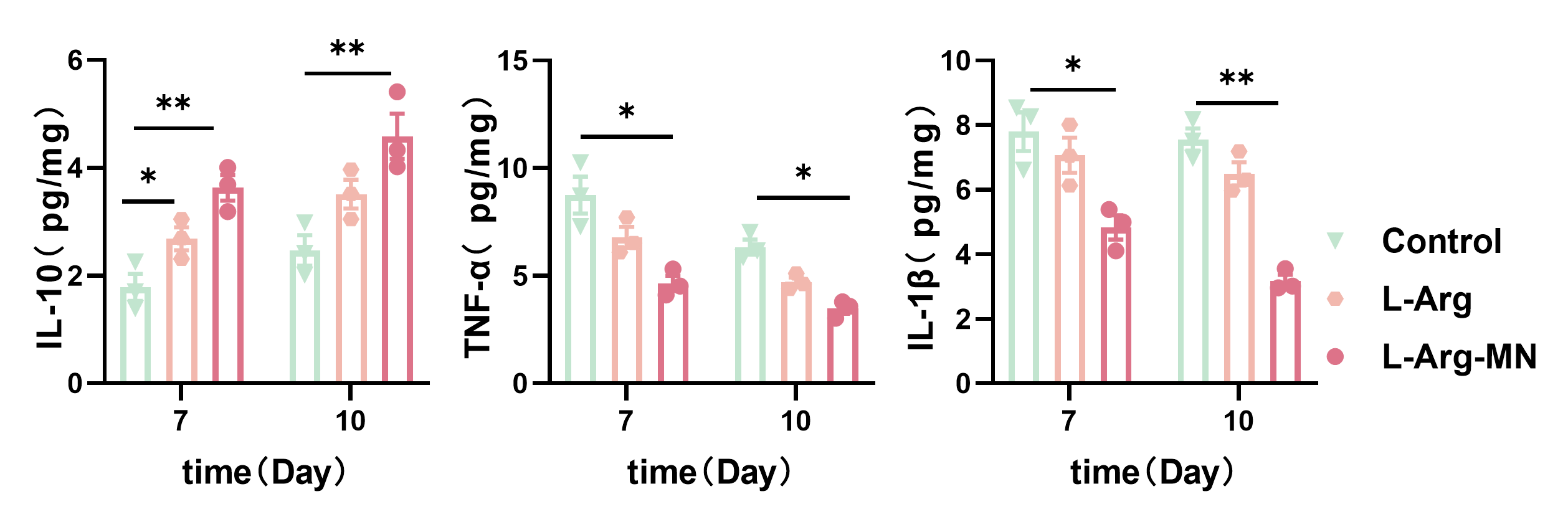


**Figure S11.** IL-10, TNF-α and IL-1β of wound tissue on days 7 and 10 by ELISA (n =3). Data are expressed as mean ± SD from at least three independent experiments. Statistical analyses were conducted using one-way ANOVA (**P* < 0.05, ***P* < 0.01).


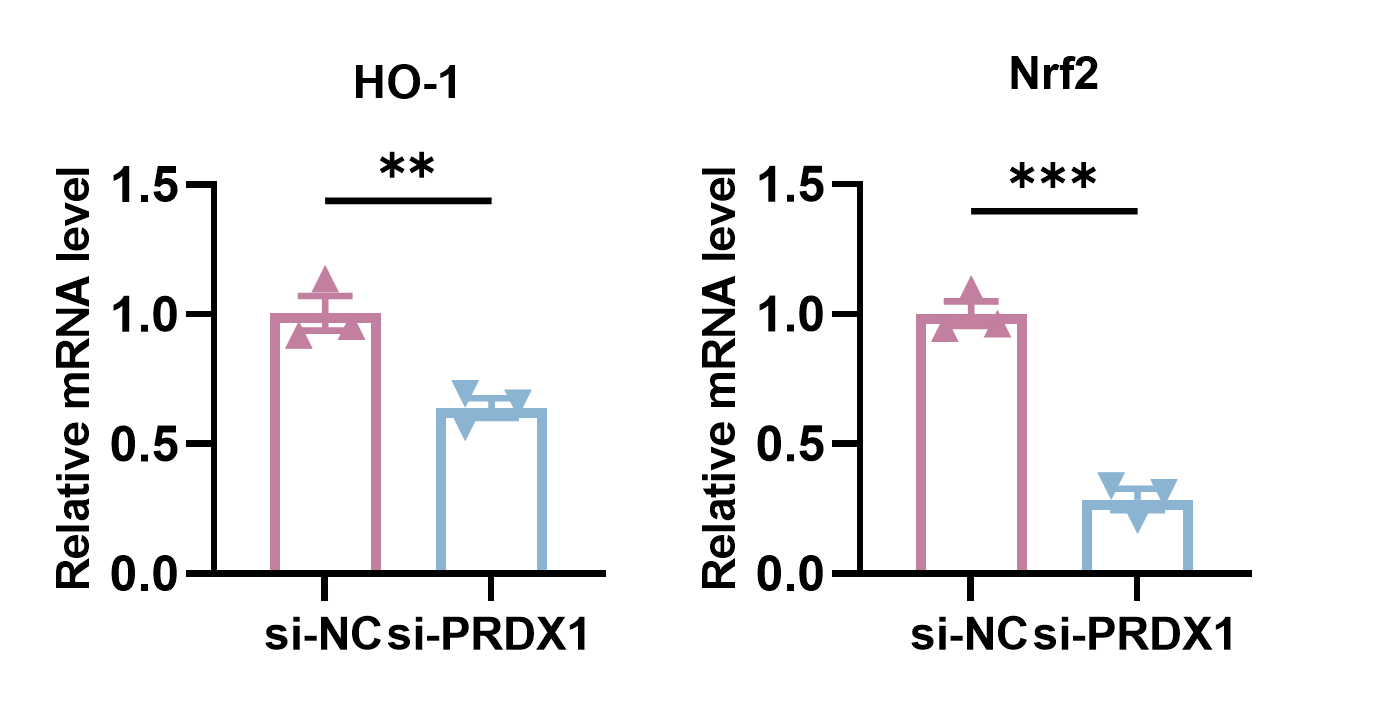


**Figure S12.** qRT-PCR analysis of mRNA levels of Nrf2, HO-1 Effect of si-PRDX1(n =3). Results are shown as mean ± SD from a minimum of three independent experiments. Statistical comparisons were performed using Student’s t-test (***P* < 0.01, ****P* < 0.001).
