# Supplementary material for: L-arginine-loaded microneedle patch enhances diabetic wound healing by regulating macrophage polarisation and mitochondrial homeostasis
Source: Regen Biomater. 2025 Sep 1;12:rbaf092. doi: 10.1093/rb/rbaf092 (PMC12493038; doi:10.1093/rb/rbaf092)
Supplement: rbaf092_Supplementary_Data [file rbaf092_supplementary_data.zip › figure.pdf]

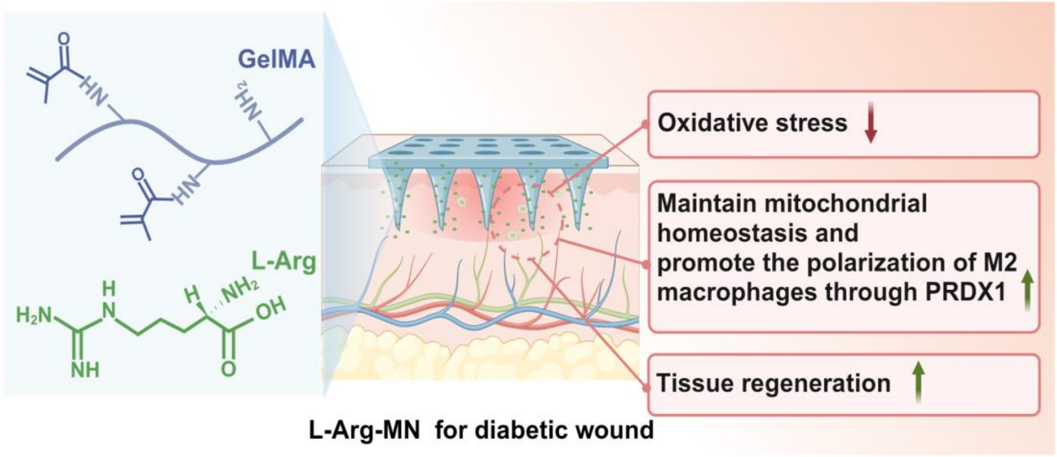
GelMA
NH2
HN
HN
O
O
L-Arg
H2N
H
N
NH
H
NH2
OH
O
Oxidative stress
Maintain mitochondrial
homeostasis and
promote the polarization of M2
macrophages through PRDX1
Tissue regeneration
L-Arg-MN for diabetic wound

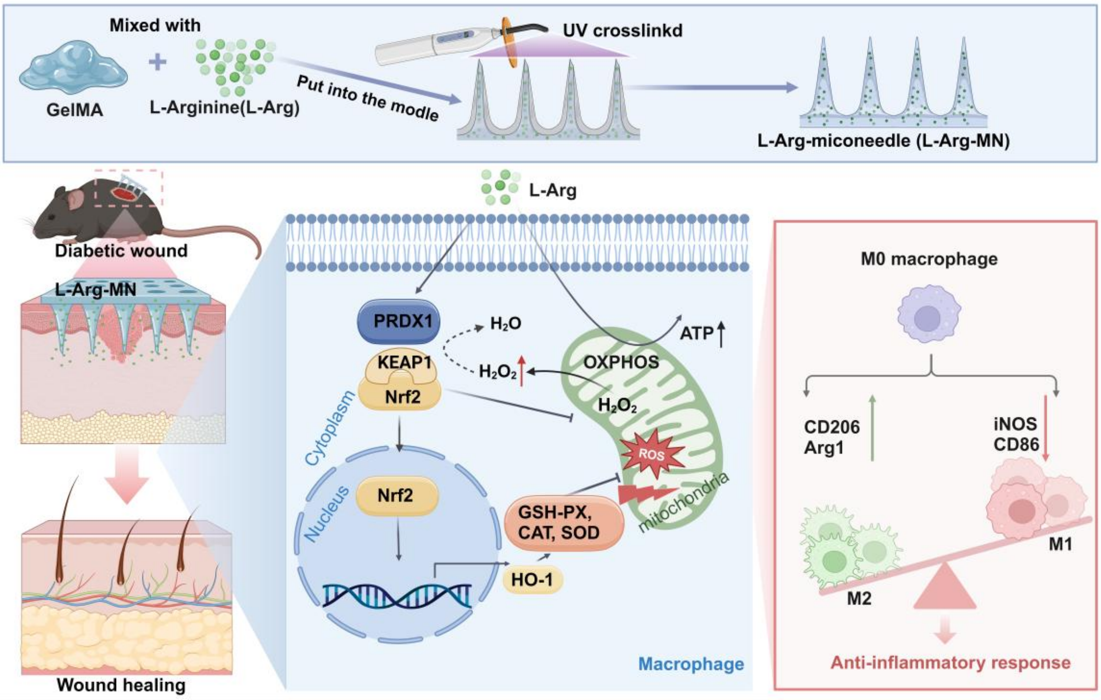

Figure 1. Schematic of how L-Arg-MN mitigates oxidative stress, regulates inflammation, and accelerates wound healing through the KEAP1-Nrf2-HO-1/ PRDX1 signalling pathway (schematic illustrations were created using BioRender: www.biorender.com).

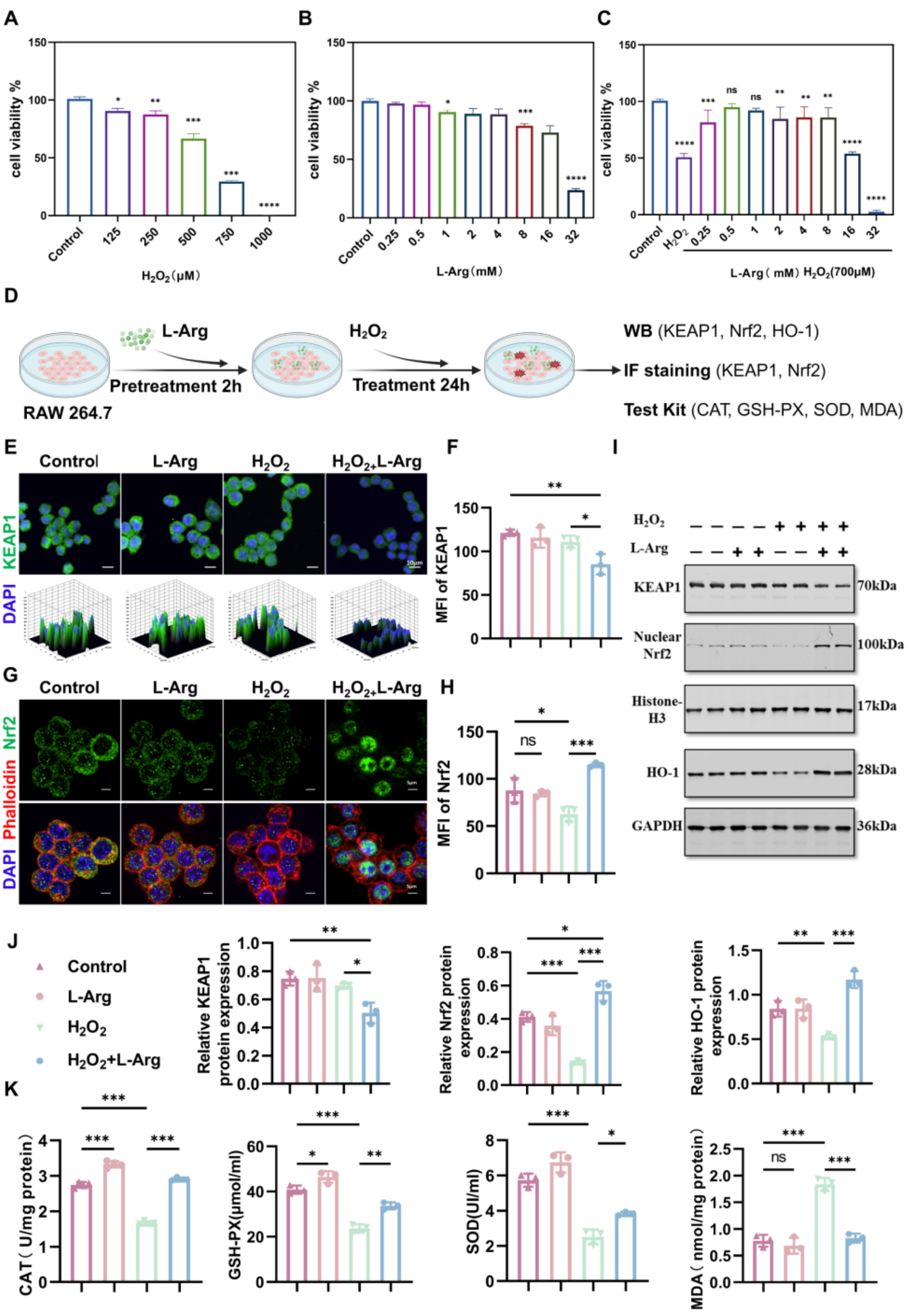

Figure 2. L-Arg attenuates H_2O_2-induced damage in RAW 264.7 macrophages by modulating the KEAP1-Nrf2-HO-1 pathway. (A) H_2O_2 dose-response (24 h); (B) L-Arg cytotoxicity (24 h); (C) L-Arg (0.25-32 mM, 2 h) pretreatment efficacy against 700 μM H_2O_2 (24 h); (D) Experimental schematic; (E-H) KEAP1/Nrf2 immunofluorescence with quantification; (I-J) Western blot of KEAP1, nuclear Nrf2 and HO-1; (K) Levels of MDA and activities of antioxidant enzymes (CAT, SOD, GSH-Px). Results are shown as mean ± SD from a minimum of three independent experiments. Statistical comparisons were performed using one-way ANOVA (**P* < 0.05, ***P* < 0.01, ****P* < 0.001; NS = not significant).

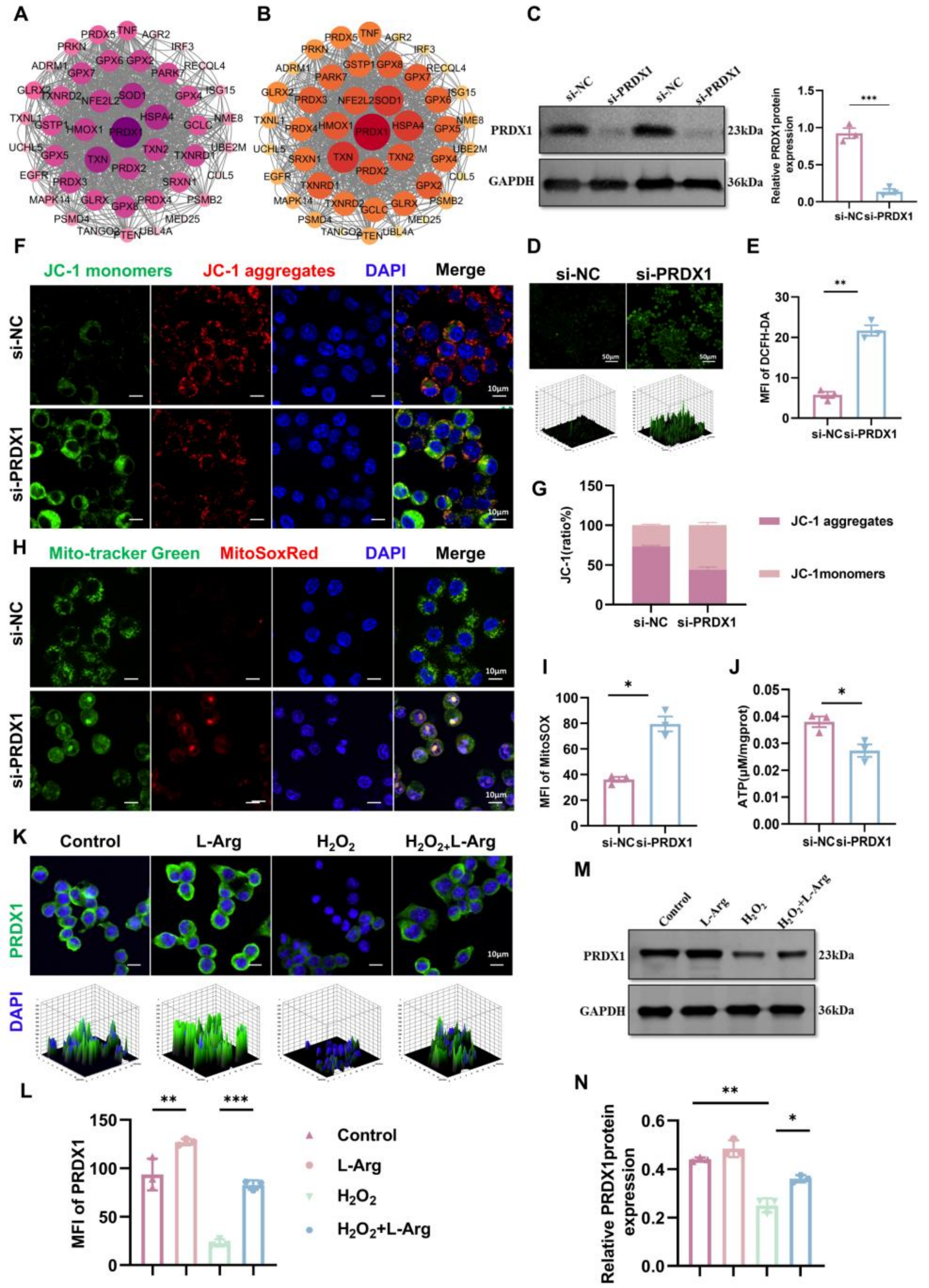

Figure 3. L-Arg upregulates PRDX1 expression and alleviates oxidative stress. (A-B) STRING database analysis of PRDX1 PPI network and oxidative damage pathway associations. (C) PRDX1 knockdown validation by WB (si-PRDX1, si-NC). (D-E) Intracellular ROS levels. (F, G) JC-1 fluorescence images and quantitative analysis showing MMP changes. (H, I) Mitochondrial superoxide production visualized by MitoSOX Red staining and quantified. (J) Quantitative analysis of ATP levels treated with si-NC and si-PRDX1. (K-L) PRDX1 immunofluorescence in H_2O_2/L-Arg-treated cells. (M-N) PRDX1 protein expression by WB. Results are shown as mean ± SD from a minimum of three independent experiments. Statistical comparisons were performed using one-way ANOVA or Student's t-test (**P* < 0.05, ***P* < 0.01, ****P* < 0.001).

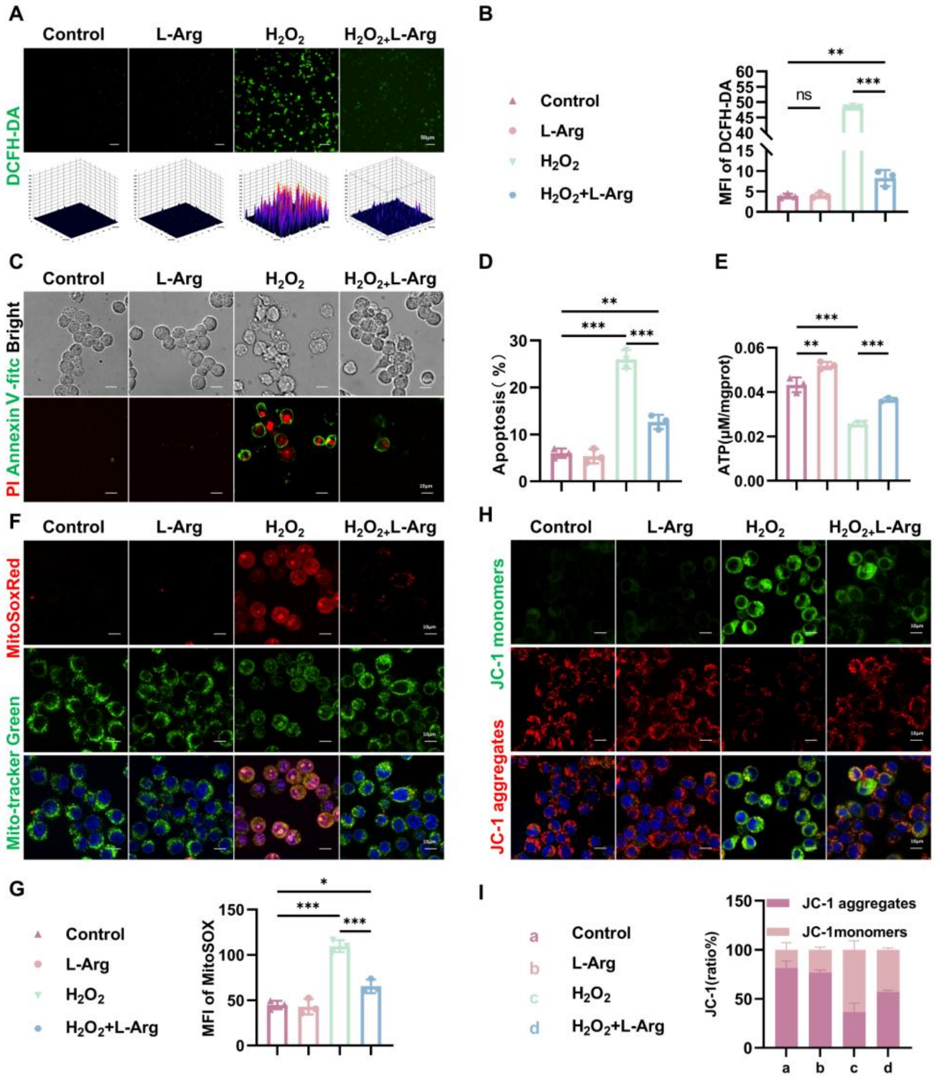

Figure 4. Oxidative stress responses in RAW264.7 macrophages. (A-B) Intracellular ROS detection using DCFH-DA fluorescence. (C-D) Apoptosis analysis. (E) ATP quantification. (F-G) MitoSOX Red staining Mitochondrial superoxide. (H-I) MMP alterations evaluated by JC-1 assay. Data are expressed as mean ± SD from at least three independent experiments. Statistical analyses were conducted using one-way ANOVA (*$P < 0.05$, **$P < 0.01$, ***$P < 0.001$).

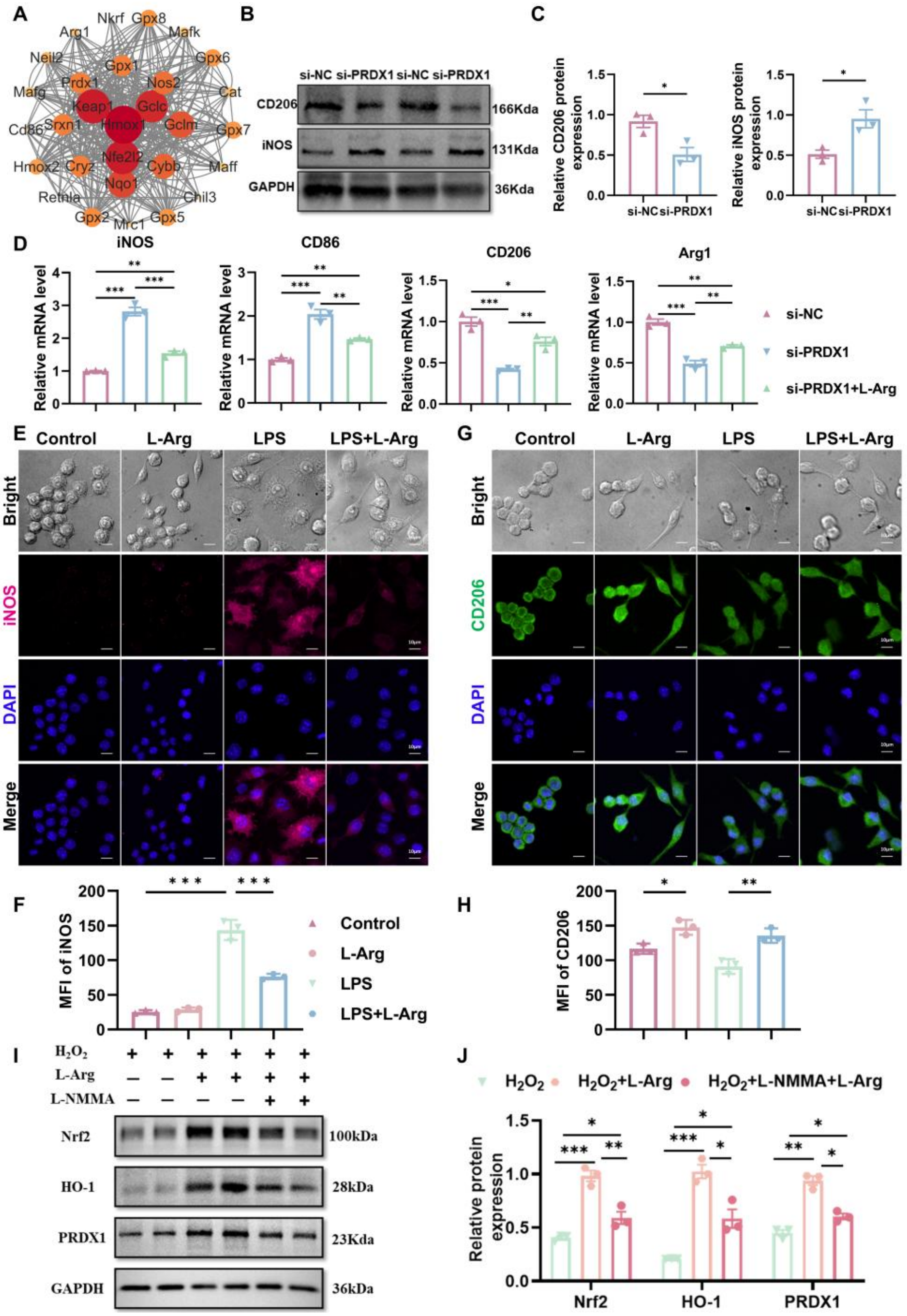

Figure 5. L-Arg modulates macrophage polarization via PRDX1. (A) STRING network analysis of PRDX1 interactions with KEAP1-Nrf2-HO-1 and polarization markers. (B-C) Western blot of CD206, iNOS in si-PRDX1 cells. (D) qRT-PCR of polarization markers with different treatments. (E-H) Immunofluorescence visualization and quantification of iNOS, CD206 in L-Arg/LPS-treated cells. (I-J) Western blot analysis of Nrf2, HO-1 and PRDX1 expression in cells under different treatments. Data are expressed as mean ± SD from at least three independent experiments. Statistical analyses were conducted using one-way ANOVA or Student's t-test (*$P < 0.05$, **$P < 0.01$, ***$P < 0.001$).

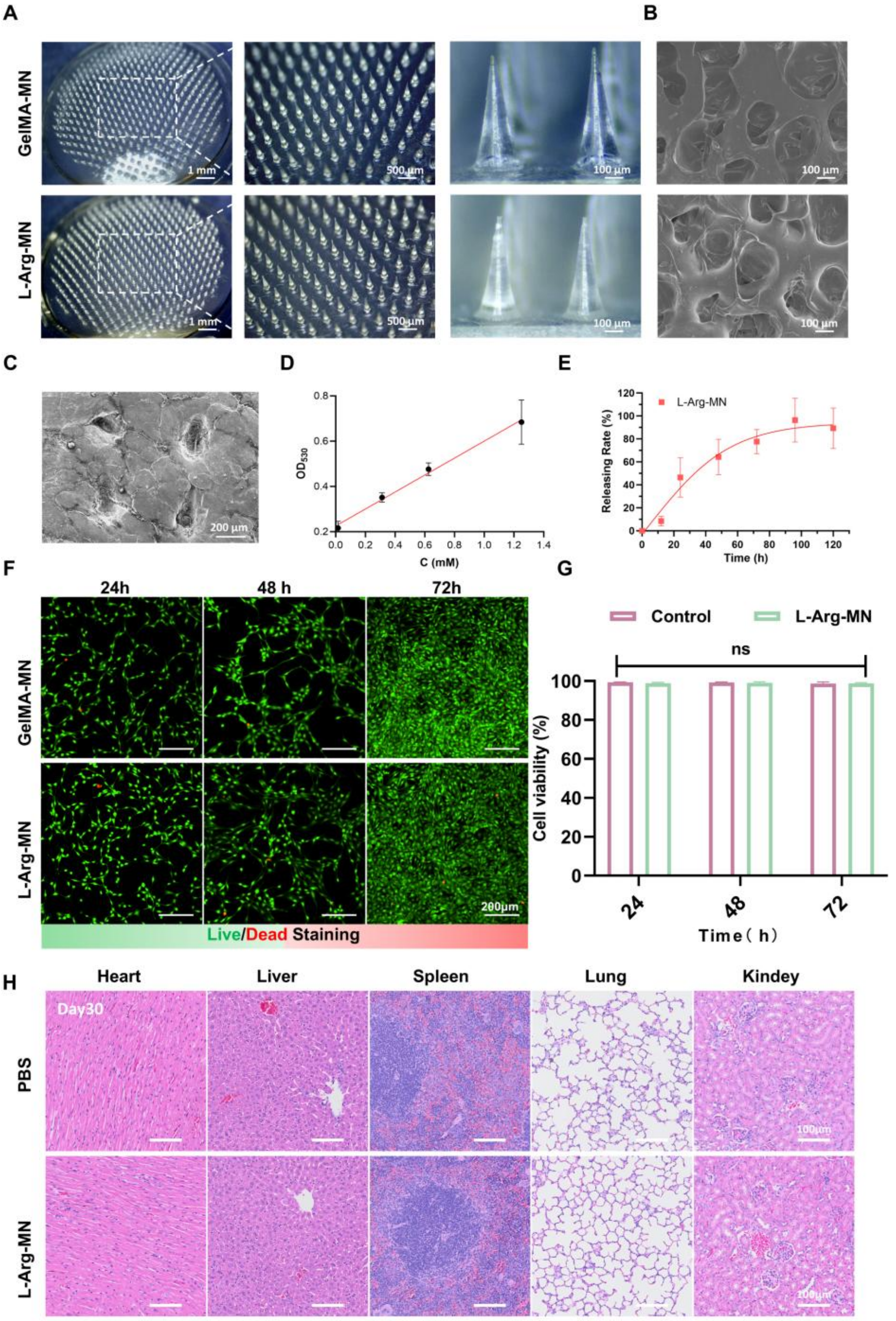

Figure 6. Characterization of L-Arg-MN. (A) Optical images of GelMA-MN vs L-Arg-MN. (B) SEM showing porous microstructure. (C) Skin penetration capability. (D) L-Arg quantification standard curve. (E) *In vitro* L-Arg release profile. (F-G) Biocompatibility assessment (cell viability). (H) H&E-stained major organs after 30-day treatment. Results are shown as mean ± SD from a minimum of three independent experiments. Statistical comparisons were performed using one-way ANOVA (**P* < 0.05, ***P* < 0.01, ****P* < 0.001, NS = not significant).

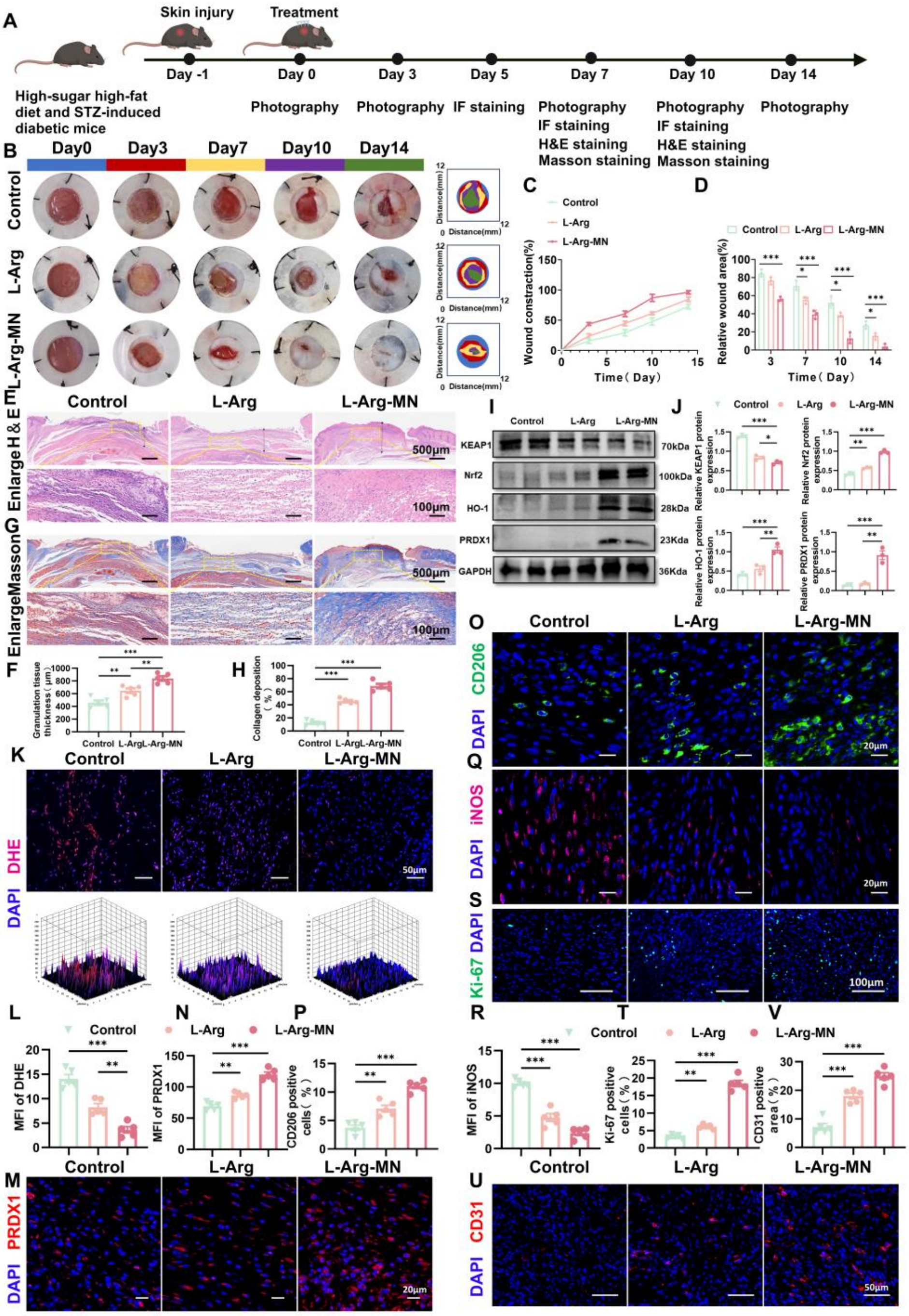

Figure 7. L-Arg-MN promotes diabetic wound healing. (A) Experimental schematic. (B) Wound appearance at indicated timepoints. (C-D) Quantification of healing rates and wound areas. (E-H) Histology (H&E and Masson's) on day 7. (I-J) WB of KEAP1/Nrf2/HO-1/PRDX1. (K-L) DHE staining (day 5) and ROS quantification. (M-N) PRDX1 immunofluorescence (day 7). (O-T) Immunofluorescence of CD206/iNOS/Ki67 (day 7). (U-V) CD31 staining (day 10). Results are shown as mean ± SD from a minimum of three independent experiments. Statistical comparisons were performed using one-way ANOVA (*$P < 0.05$, **$P < 0.01$, ***$P < 0.001$).
